# Seven years in formalin: the burden of metabarcoding mesozooplankton preserved in buffered formalin from the northern Adriatic Sea

**DOI:** 10.1093/plankt/fbag066

**Published:** 2026-08-21

**Authors:** Elettra Chiarabelli, Alessandra de Olazabal, Alenka Goruppi, Sara D'Ambros Burchio, Marco Sollitto, Valentina Tirelli, Alberto Pallavicini

**Affiliations:** Department of Life Sciences, University of Trieste, Via L. Giorgieri 5, Trieste 34127, Italy; National Institute of Oceanography and Applied Geophysics - OGS, Via A. Piccard 54, Trieste 34151, Italy; National Institute of Oceanography and Applied Geophysics - OGS, Via A. Piccard 54, Trieste 34151, Italy; CoNISMa, Piazzale Flaminio 9, Roma 00197, Italy; Department of Life Sciences, University of Trieste, Via L. Giorgieri 5, Trieste 34127, Italy; CoNISMa, Piazzale Flaminio 9, Roma 00197, Italy; Department of Biology, University of Florence, Via Madonna del Piano 6, Sesto Fiorentino, 50019, Italy; National Institute of Oceanography and Applied Geophysics - OGS, Via A. Piccard 54, Trieste 34151, Italy; NBFC—National Biodiversity Future Center, Piazza Marina 61, Palermo 90133, Italy; Department of Life Sciences, University of Trieste, Via L. Giorgieri 5, Trieste 34127, Italy; National Institute of Oceanography and Applied Geophysics - OGS, Via A. Piccard 54, Trieste 34151, Italy; CoNISMa, Piazzale Flaminio 9, Roma 00197, Italy; NBFC—National Biodiversity Future Center, Piazza Marina 61, Palermo 90133, Italy

**Keywords:** formalin-fixed mesozooplankton, metabarcoding, DNA, Gulf of Trieste, historical collection, COI, 18sv4, 18sv9

## Abstract

Historical mesozooplankton collections preserved in formalin are valuable archives for reconstructing past biodiversity, but formalin-induced DNA damage has long limited their molecular use. We evaluated the feasibility of metabarcoding on mesozooplankton samples collected in 2017 from the northern Adriatic Sea and stored in 4% buffered formalin for approximately seven years at room temperature and dark conditions. We applied a hot alkaline lysis protocol to improve DNA recovery from formalin-fixed material and tested three metabarcoding markers differing in amplicon length and taxonomic resolution (COI, 18SV4, and 18SV9), using a bioinformatic pipeline adapted for highly degraded templates. The three markers performed differently. COI retained the strongest metazoan signal, while 18SV9 recovered the broadest metazoan diversity and showed the highest concordance with morphology. By contrast, 18SV4 was strongly biased toward non-metazoan amplification, with fungal reads dominating several samples. Of the 86 taxa identified by stereomicroscopy, COI recovered 16 taxa and 18SV9 recovered 59, with only nine shared between markers. These results show that samples preserved in buffered formalin for 7 years can still yield informative metabarcoding data, although taxonomic recovery is strongly shaped by marker choice.

## INTRODUCTION

Zooplankton communities are widely used as indicators of ecosystem condition, climate-driven regime shifts and biological introductions (Bucklin *et al.,* 2010; Lindeque *et al.,* 2013; Menéndez *et al.,* 2013; Pierson *et al.,* 2021). Molecular approaches have increasingly contributed to understanding zooplankton diversity, revealing cryptic species, refining taxonomic boundaries and improving the detection of rare or morphologically indistinguishable taxa (Amaral-Zettler *et al.,* 2009; de Vargas *et al.,* 2015; Stefanni *et al.,* 2018; Bucklin *et al.,* 2021; Di Capua *et al.,* 2023). Historical zooplankton samples collected through monitoring programs and long term ecological research represent an archive for reconstructing past biodiversity patterns and ecological trajectories; nonetheless, most have been preserved in formalin, a fixative known to compromise nucleic acids. Formalin fixation induces DNA-protein cross-links, depurination, base modification and extensive fragmentation (e.g. Gilbert *et al.,* 2007; Campos and Gilbert, 2012; Hahn *et al.,* 2022). Such chemical alterations severely reduce the quality and quantity of recoverable DNA, complicating PCR amplification and increasing the risk of artifactual sequences. Because PCR amplification of fragmented DNA is particularly susceptible to sequencing artifacts, several authors have recommended either performing deep amplicon sequencing to compensate for template damage (Appleyard *et al.,* 2021; Hahn *et al.,* 2022) or avoiding amplicon approaches altogether in favor of whole-genome sequencing (WGS) of degraded templates (Burrell *et al.,* 2015). However, WGS is considerably more challenging when applied to multispecies assemblages such as bulk zooplankton samples, as they require extensive sequencing effort and computationally demanding post-processing to disentangle hundreds of genomes from mixed templates. Acknowledging these constraints, we explored an amplicon-based strategy, as it offers a practical and informative approach for investigating highly complex formalin-preserved zooplankton samples. Formalin preservation does not always prevent molecular analysis. Bucklin and Allen (2004) recovered mitochondrial sequences from the euphausiid *Meganyctiphanes norvegica* after 25 years in buffered formalin. Hou *et al.* (2021) and Appleyard *et al.* (2022) applied DNA barcoding to individual formalin-fixed fish larvae. Heindler *et al.* (2018) used metabarcoding on stomach and hindgut samples from historical fish specimens; Wietz *et al.* (2022) applied it to preserved marine microbial communities. Marine bulk metabarcoding is sensitive to sample processing, extraction chemistry, marker choice, PCR conditions and reference database completeness (van der Loos and Nijland, 2021). These studies, however, differ from our design: most use single specimens or tissues, or focus on microbial or mixed plankton communities rather than bulk mesozooplankton assemblages. The peer-reviewed study closest to community-level formalin metabarcoding is Shiozaki *et al.* (2021), although its strongest validation concerned microplankton/diatom communities rather than bulk mesozooplankton. Albaina *et al.* (2026) is directly relevant to formaldehyde-preserved zooplankton time series, though currently available only as a preprint. Thus, the open question is how much taxonomic information bulk mesozooplankton assemblages retain after years in formalin, how marker choice shapes that recovery, and how molecular results compare with morphology.

To address this gap, we applied a multi-locus and a multi-marker, amplicon-based strategy to mesozooplankton samples collected in the Gulf of Trieste (Italy) and preserved for a long time in formalin. We combined a DNA extraction protocol optimized for cross-link reversal, three primer sets differing in amplicon length and taxonomic resolution, and a bioinformatic pipeline adapted to chemical damage. Recovering ecological information from formalin-preserved zooplankton archives matters beyond the methodological results here: these collections document past biodiversity and community change over decades. Our framework offers one practical step toward using them. Similar archives sit unused in collections worldwide, and this work shows one possible way to start extracting data from them.

## MATERIAL AND METHODS

### Zooplankton sampling

**Fig. 1:**
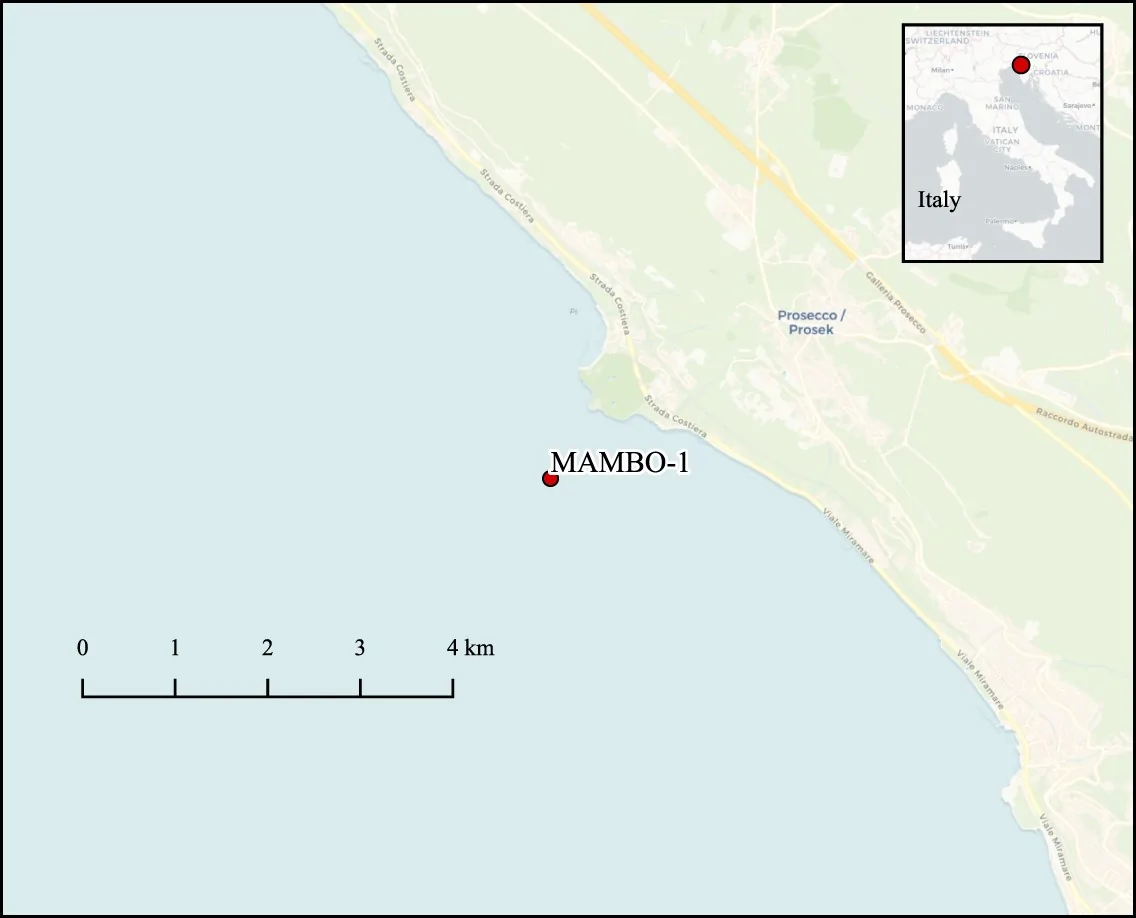
Location of the meteo-oceanographic buoy MIRAMARE (MAMBO-1; 45°41′51.7″ N, 13°42′29.9″ E) in the Gulf of Trieste, northern Adriatic Sea. The inset map shows the position of the study area within the Italian peninsula.

Twelve mesozooplankton community samples were collected monthly in 2017 by OGS staff at the meteo-oceanographic buoy MIRAMARE (MAMBO-1; 45°41′51.7″ N, 13°42′29.9″ E), located less than 400 m from the Long Term Ecological Research (LTER) Gulf of Trieste station (Fig. 1) and part of the ICOS Integrated Carbon Observation System network. Samples were collected by vertical hauls using a 200 μm mesh WP2 plankton net, towed from 3 m above the bottom to the surface. After collection, samples were immediately fixed in 4% buffered formalin.

### Morphological identification

For the morphological analysis, carried out using a dissecting stereomicroscope (Leica 165C-120×, Olympus SZX16-115×), samples were concentrated on a 200 μm mesh net rinsed to remove formaldehyde and resuspended in filtered seawater. A subsample of at least 1000 individuals was analysed, and taxa identification was carried out to the lowest taxonomic level following taxonomic literatures (Avancini *et al.,* 2006; Boltovskoy, 1999; Castellani and Edwards, 2017; Nishida, 1985; Razouls *et al*., 2005-2025; Rose, 1933; Tregouboff and Rose, 1957). Abundances were expressed as the number of individuals per cubic metre (ind/m^3^). After this analysis, each sample was resuspended in seawater-buffered formaldehyde solution (4% final concentration) and then stored at dark and room temperature, until the molecular analysis was performed. Roughly all the samples remained in formalin for seven years.

### DNA extraction and PCR amplifications

Sieving the samples on a 200 μm mesh, formalin was removed from the samples, zooplankton was quickly washed with deionized water and then stored in 70% ethanol at −20°C until further processing. Prior to the extraction, we discharged all ethanol and pulverized the bulk using a stainless steel bead in the VWR® Bead Mill Homogenizer (Avantor™). For genomic DNA extraction, we used a hot alkaline lysis (0.1 M NaOH, 1% SDS) following the instructions reported from Hahn  *et al.*  (2022) and then we incubated the samples at 98°C for 30 minutes. After neutralizing the pH with 0.1 M HCl, we processed 25 μL of the obtained lysate using the E.Z.N.A.® Mollusc DNA kit (Omega Bio-Tech), following the manufacturer’s instructions. For the digestion with the Proteinase K Solution we incubated the samples at 65°C for 1 hour, as increasing the incubation temperature could improve the effectiveness of the treatment (Straube *et al.,* 2021). We quantified the extracted DNA using Qubit fluorometer with the Qubit™ double-stranded DNA (dsDNA) HS Assay Kit and the Qubit™ single-stranded DNA (ssDNA) Assay Kit (Thermo Fisher Scientific™). A blank control was processed alongside the samples to check for potential contamination during DNA extraction. We performed PCR amplification for each sample, testing three primer sets: one targeting mitochondrial DNA (COI) and two targeting nuclear DNA (18SV4 and 18SV9) (Table I).

**Table I:** Primer used for the PCR amplifications

Target region	Primer	Sequence (5′ - 3′)	Reference
COI (∽313 bp)	*mlCOIintF*	GGWACWGGWTGAACWGTWTAYCCYCC	Leray *et al.* (2013)
	*CrustDR1*	TAAACYTCAGGRTGACCRAARAAYCA	Knox *et al.* (2012)
18SV9 (∼149 bp)	*1389F*	TTGTACACACCGCCC	Amaral-Zettler *et al.* (2009)
	*1510R*	CCTTCYGCAGGTTCACCTAC	
18SV4 (∼380 bp)	*TAReukFWD1*	CCAGCASCYGCGGTAATTCC	Stoeck *et al.* (2010)
	*TAReukREV3*	ACTTTCGTTCTTGATYRA	

PCR amplifications were carried out in a total volume of 25 μL, containing 12.5 μL of 2× PCRBIO HS *Taq* Mix (PCRBIOSYSTEMS), 0.8 μL of both forward and reverse primers (10 μM), 1.25 μL of EvaGreen^Ⓡ^ dye (Biotium), and 1 μL of genomic DNA.

We selected three commonly used primer sets to characterize the zooplankton community. The 18SV9 region is often adopted in marine metabarcoding studies (Bucklin *et al.,* 2019; Schroeder *et al.,* 2020; Stefanni *et al.,* 2018; Bucklin *et al.,* 2019; Schroeder *et al.,* 2020;) because it is a short hypervariable region (~150 bp), enabling broad amplification across diverse eukaryotes. We also incorporated the 18SV4 region (~380 bp), as several studies indicate that 18SV9 may not always provide optimal resolution for zooplankton biodiversity (Blanco-Bercial, 2020), and also Questel *et al.* (2021) demonstrated that 18SV4 can improve taxonomic resolution in marine communities. To increase taxonomic resolution and reduce primer-specific bias, we also included a mitochondrial COI primer pair targeting the 313 bp “P2” region (Schroeder *et al.,* 2021), as COI has repeatedly been shown to outperform 18S in species-level discrimination. Clarke *et al.* (2017) reported up to a threefold increase in species-level assignments with COI compared to 18S.

Each primer set was run with a specific thermal cycling profile. For the COI fragment, the protocol included an initial denaturation at 95°C for 1 min, followed by seven cycles of 95°C for 15 s, 46°C for 20 s, and 72°C for 3 s, then 45 additional cycles with an annealing temperature of 54°C. For the 18SV4, 50 cycles of 95°C for 15 s, 48°C for 20 s, and 72°C for 30 s, and for the 18SV9, 50 cycles 95°C for 15 s, 50°C for 20 s, and 72°C for 30 s.

All samples were purified using the Mag-Bind™ Total Pure NGS kit (Omega Bio-Tech), following the manufacturer’s instructions. We then prepared the samples for sequencing with a secondary PCR, using the same reagent volumes as in the primary PCR, primers with Illumina TruSeq barcoded sequences and 2 μL of the first PCR product as template. We purified and quantified all samples again before pooling them into an equimolar library for sequencing (Mag-Bind™ Total Pure NGS kit and Qubit™ dsDNA HS Assay Kit). The library was sequenced on an Illumina Novaseq6000 platform using a paired-end run (2 × 250 bp). The LAGE (Laboratory of Genomics and Epigenomics) at Area Science Park (Trieste) carried out the sequencing of the samples.

### Sequence data processing and taxonomic assignment

We used QIIME2 (version 2024.5; Bolyen, *et al*., 2019) to quality control, filter and trim raw paired-end reads with the cutadapt trim-paired plugin. We discarded sequences shorter than 50 bp after trimming. We then used the DADA2 denoise-paired plugin (Callahan *et al*., 2016) to perform denoising, dereplication, chimera filtering, and feature table construction. Starting from a threshold of 8, we progressively lowered the –p-max-ee-f and –p-max-ee-r parameters until reaching a setting that retained at least ~ 65% of the reads per sample. For COI and 18SV9, we set the -p-max-ee-f and –p-max-ee-r parameters to 4, and to 3 for 18SV4. To perform OTU clustering, we exported representative sequences from QIIME2 and processed them externally using VSEARCH (Rognes *et al.,* 2016). Rather than applying the commonly used distance-based greedy clustering (DGC) approach, we used the abundance-based greedy clustering (AGC) algorithm, which better accommodates sequencing noise and abundance imbalances typical of formalin-derived datasets. We then evaluated multiple similarity thresholds across all primer sets to assess cluster stability and the sensitivity of AGC to formalin-related artifacts. This optimization step allowed us to determine the most appropriate identity threshold for each marker prior to downstream taxonomic assignment. The feature-level table was collapsed into an OTU-level abundance table in QIIME2 using a custom Python script and the VSEARCH-generated sequence mappings. The taxonomy was assigned using Naive Bayes classifiers trained on different reference databases: MetaZooGene for COI (Bucklin *et al.,* 2021) and SILVA for 18S (Quast *et al.,* 2012). To validate the taxonomic assignments, we also performed BLAST searches of the clustered centroid sequences against the NCBI nucleotide database. All three taxonomic table results were manually checked. For each mesozooplankton taxon, we verified the assignments against known species distributions in the Adriatic Sea (Razouls *et al.,* 2005-2025; Word Register of Marine Species—online databases). Taxa identified through morphological analysis were also examined to determine whether corresponding reference sequences were available. We accepted OTU-level taxonomic assignments when the percent identity exceeded 97%. When the identity ranged between 94% and 97%, we considered the assignment less reliable and added a “cf.” prefix to indicate uncertainty. For matches between 94% and 85% identity, we could not confidently assign species-level identity and therefore limited the classification to the family level. For identities below 85%, sequences were discarded.

### Molecular analysis: taxonomic composition and relative abundance

Given the combined effects of formalin preservation, extensive DNA fragmentation, degradation, and the need for high PCR cycle numbers to obtain amplifiable material, standard eDNA and metabarcoding analytical workflows are not applicable to these samples. We therefore adopted a modified analytical strategy designed to accommodate formalin-induced damage and distortion and to allow a primer performance rather than inference of ecological patterns. We calculated the relative abundance of reads per Kingdom by dividing the number of reads assigned to a given Kingdom by the total read count. After this initial exploratory assessment, downstream summaries of taxonomic composition were based on the cleaned datasets. Animalia phylum composition was quantified for all datasets, and phylum-level relative abundances were visualized using 100% stacked bar plots. For each primer, we also quantified the number of OTUs and total reads assigned to Animalia by summing monthly read counts across taxonomically annotated entries. We generated taxon-level summary figures for each Animalia phylum using a custom R workflow (dplyr, tidyr, ggplot2, VennDiagram, and gridExtra, R Core Team (2021). The script read the cleaned species table, standardized taxonomic labels, and calculated presence across methods. For each phylum, the workflow produced three panels: the number of detected taxa per method, a three-way Venn diagram showing shared taxa, and a plot of assignment identity for COI and 18SV9.

## RESULTS

The concentration of genomic DNA extracted from the samples ranged from 0.39 ng/μL to 6.98 ng/μL for dsDNA, and from 1.25 ng/μL to 25.14 ng/μL for ssDNA (Table II). Sequencing depth was highest for COI, with 13 013 662 raw paired-end reads, followed by 18SV4 (11 334 580) and 18SV9 (9 102 269). Given the severe distortions introduced by formalin preservation, we applied the adapted bioinformatic workflow outlined in Fig. S1.

**Table II:** Quantification of genomic DNA with Qubit™ dsDNA HS Assay Kit and the Qubit™ ssDNA DNA Assay Kit (Thermo Fisher Scientific™)

Sample	Stock concentration dsDNA	Units	Stock concentration ssDNA	Units
December	2.64	ng/μL	14,24	ng/μL
November	0.48	ng/μL	1,25	ng/μL
October	0.42	ng/μL	1,40	ng/μL
September	2.57	ng/μL	16,60	ng/μL
August	3.58	ng/μL	11,98	ng/μL
July	0.39	ng/μL	2,25	ng/μL
June	0.73	ng/μL	2,64	ng/μL
May	0.88	ng/μL	5,13	ng/μL
April	0.67	ng/μL	3,04	ng/μL
March	2.38	ng/μL	10,98	ng/μL
February	3.13	ng/μL	16,76	ng/μL
January	6.98	ng/μL	25,14	ng/μL

**Fig. 2:**
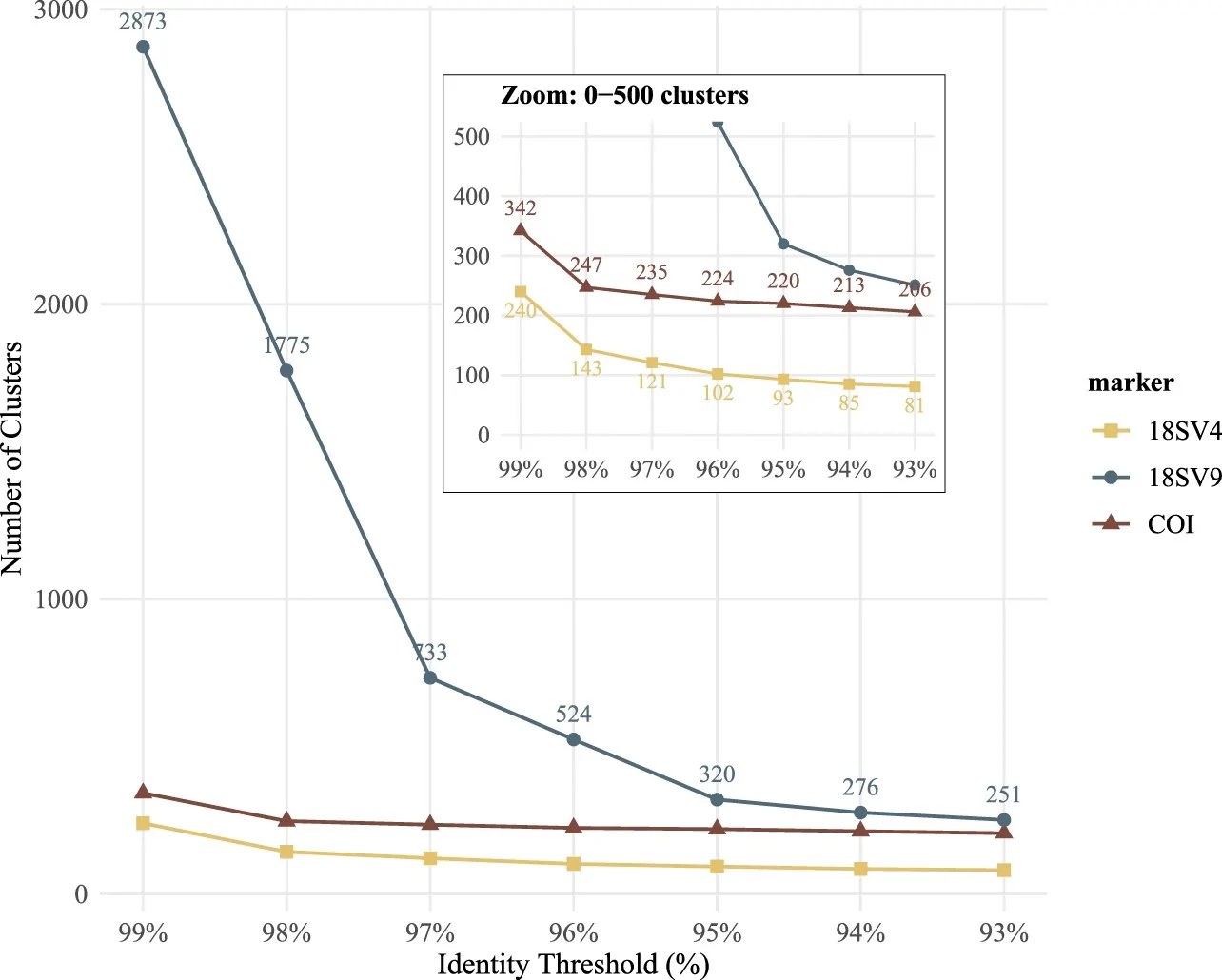
Number of AGC clusters recovered across pairwise identity thresholds (0.93–0.99) for the three markers (18SV4, 18SV9, COI). Each line shows how cluster counts decrease as similarity cutoffs become more permissive, illustrating marker-specific sensitivity to sequencing noise and formalin-related artifacts.

After stringent quality filtering, we retained 9 295 440 reads for COI, 9 274 737 for 18SV4, and 6 580 631 for 18SV9. The denoising step yielded 1482 ASVs for COI, 1053 for 18SV4, and 3696 for 18SV9. For each marker, we quantified AGC clusters across identity thresholds (0.99–0.93) to evaluate cluster stability (Fig. 2). For 18SV4, we observed a decline from 240 to 81 clusters, identifying 93 clusters at the 95% threshold. For COI, we recorded a decrease from 342 at 99% to 206 at 93%, selecting the 94% threshold (213 clusters). For the shorter 18SV9 fragment, we found that cluster counts dropped from 2873 to 251, with the 96% threshold selected for downstream analyses.

### Taxonomic composition and relative abundance

**Fig. 3:**
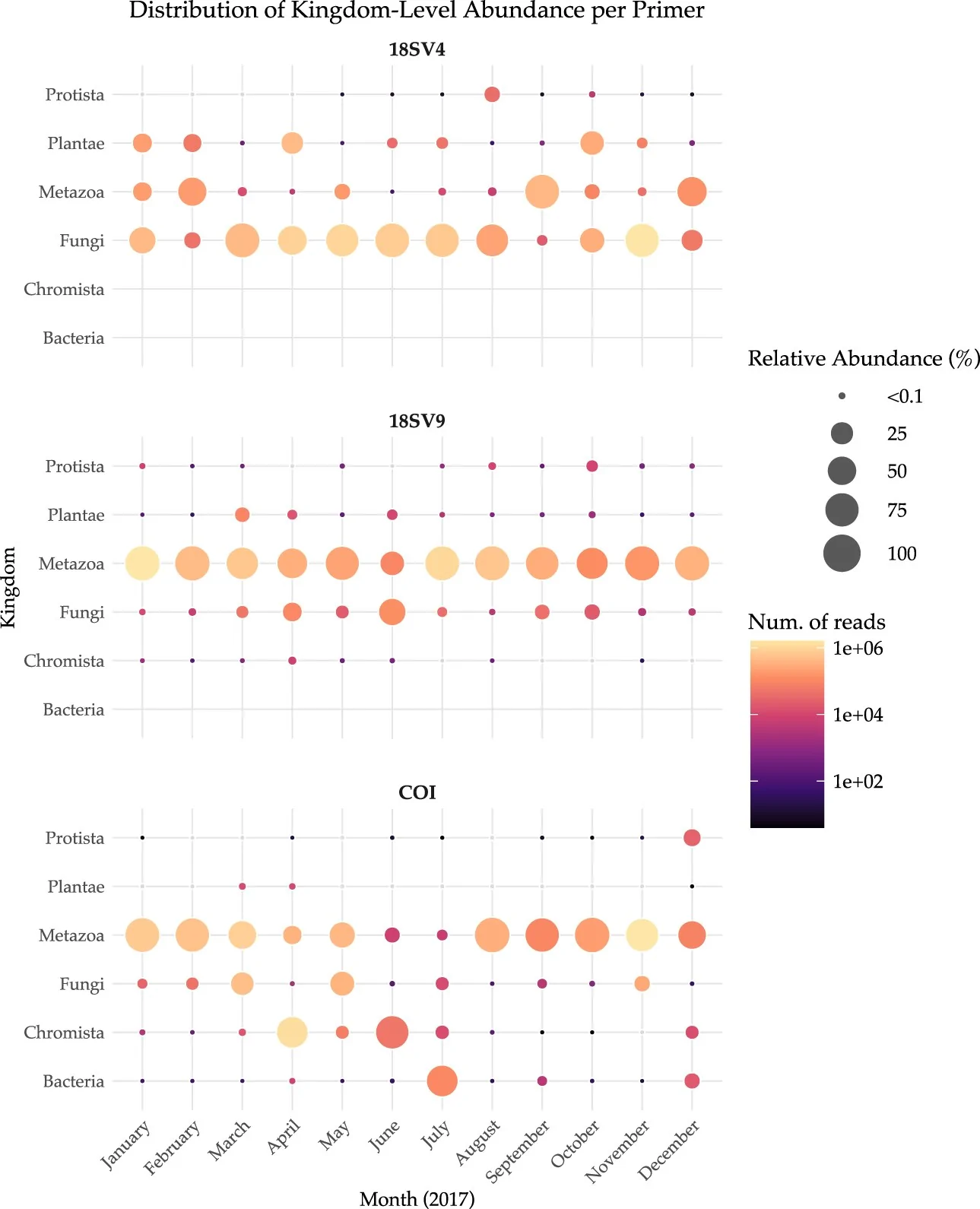
Relative abundance and read counts of taxonomic kingdoms detected per month, for each genetic marker (18SV4, 18SV9, COI) in 2017. Bubble size indicates the relative abundance (%) of each kingdom within a given month and marker; bubble colour indicates the number of reads (log-scale). Kingdoms with fewer than 0.1% relative abundance are shown as minimal-size points.

**Fig. 4:**
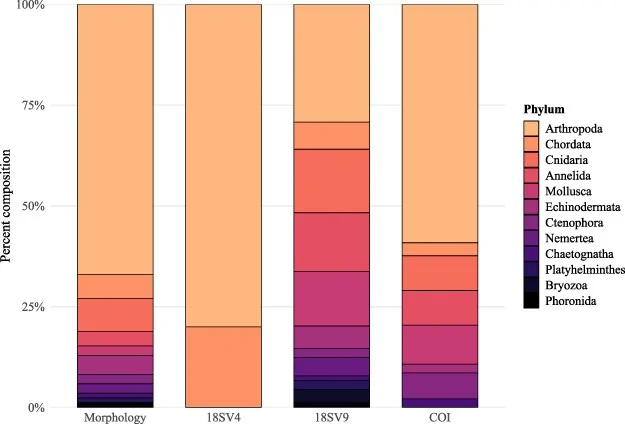
Relative abundance of metazoan phyla in the four datasets. The 100% stacked barplot presents the proportional contribution of each metazoan phylum within each dataset, based on phylum-level read totals for molecular markers and specimen counts for morphology.

Patterns of taxonomic composition differed markedly across primers. COI was strongly dominated by Animalia, with monthly contributions typically exceeding 80–95% of all reads, except in late spring and early summer when a temporary decline was observed (Fig. 3, Table SI). For 18SV4, Fungi showed the highest contributions across several months (often > 80–90%), while Animalia displayed a pronounced variability, ranging from < 1% to > 60%. 18SV9 sequences mirrored the dominance of Animalia as in COI yet showcased sharper increases in Fungi (up to > 50% in May) (Fig. 3, Table SI). Fungal OTUs were dominated by Basidiomycota, with additional contributions from Ascomycota, highlighting a strong fungal bias in the 18SV4 data compared to the other markers. Coherently with the kingdom-level patterns, the four datasets showed marked differences in metazoan phyla relative abundance (Fig. 4). The three markers differed markedly in their ability to recover metazoan signal (Table SII): COI produced 214 OTUs, of which 133 (≈62%) corresponded to Animalia, accounting for ~ 56% of all reads. The 18SV4 dataset showed much poorer metazoan representation, with only 18 metazoan OTUs (≈36%) and ~ 14% of reads assigned to Animalia. In contrast, 18SV9 yielded the strongest metazoan signal, recovering 367 metazoan OTUs (≈70% of the total) and ~ 88% of all reads. Overall, these patterns indicate substantial primer-specific differences, with COI and 18SV9 effectively capturing metazoan diversity, whereas 18SV4 showed a much weaker metazoan signal and was strongly enriched in non-metazoan taxa. Within the 18SV4 dataset, fungal OTUs were mainly represented by Agaricomycetes, which accounted for 11 of the 19 fungal OTUs identified, corresponding to 22% of all V4 OTUs and 50.5% of the total reads. For this reason, 18SV4 was excluded from the comparison with morphology, and the coverage analysis was restricted to COI and 18SV9.

### Holoplankton

#### Arthropoda

Arthropods showed the highest richness among all phyla. Morphology detected 57 taxa, the 18SV9 region detected 60 taxa, and COI detected 41 taxa, with only nine taxa shared by all three methods. Each method contributed a substantial number of exclusive detections, particularly 18SV9 (17 taxa) and COI (17 taxa). Most detections belonged to Copepoda (of which 57 taxa were identified at species level), which appeared extensively across morphology and 18SV9, while COI included a more limited subset. The non-indigenous species *Pseudodiaptomus marinus* Sato, 1913 was detected by morphology and 18SV9 but absent in COI.

Malacostraca, including amphipods, euphausiids, mysids, ostracods, and several decapod larvae, appeared unevenly across methods, with many taxa present in morphology and 18SV9 and fewer detected by COI. Two non-indigenous amphipods occur in the dataset: *Grandiderella japonica* Stephensen, 1938, and *Jassa slatteryi* Conlan, 1990, both detected exclusively by 18SV9.

**Fig. 5:**
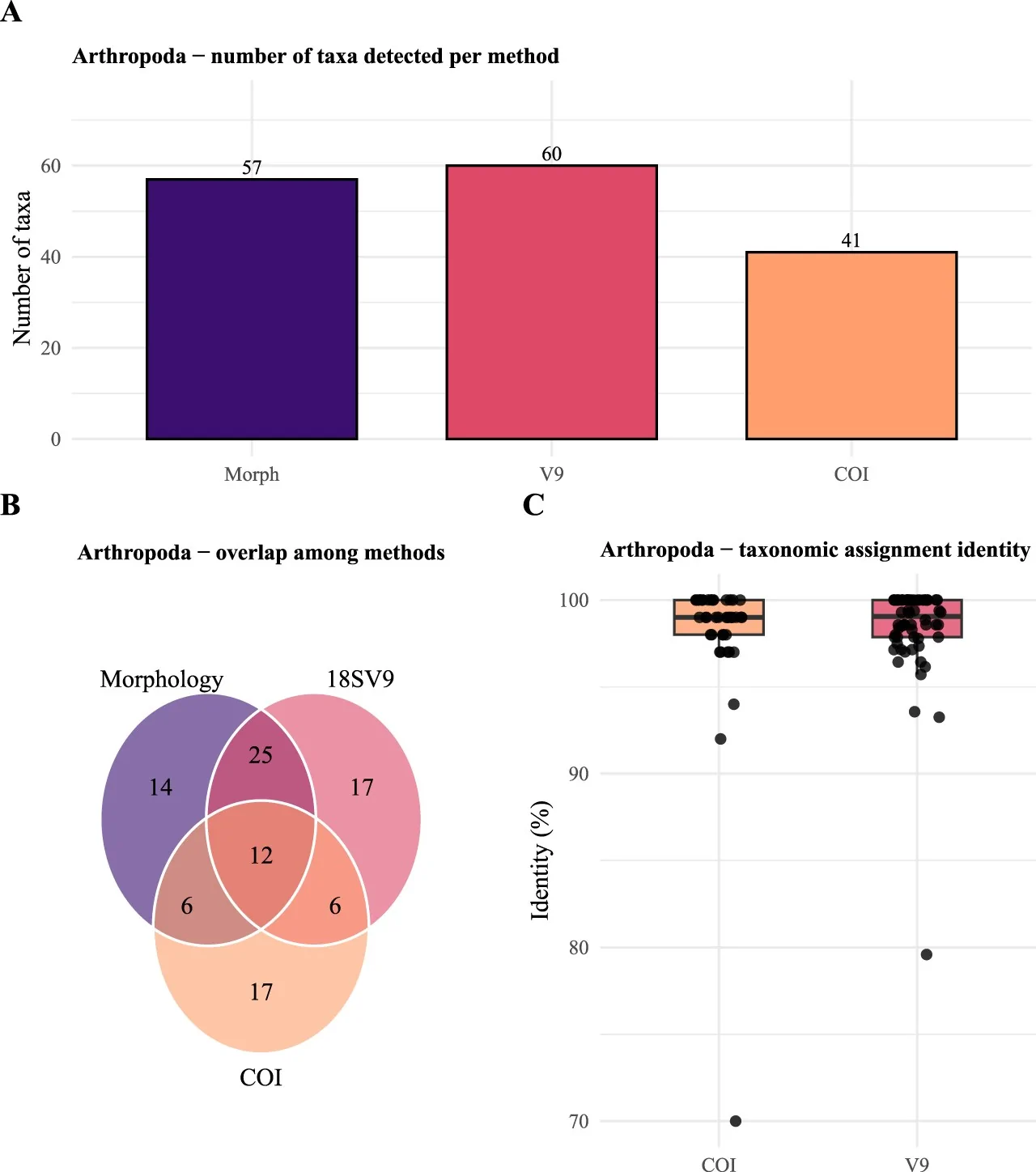
Comparison of morphological and molecular approaches for assessing Arthropoda diversity. Bar chart showing the total number of Arthropoda taxa detected by each method: Morphology (morph), 18S rRNA V9 region (V9), and cytochrome *c* oxidase subunit I (COI). Venn diagram illustrating the shared and unique Arthropoda taxa detected by morphology, 18SV9, and COI methods. The overlapping areas indicate the number of taxa identified by multiple methods, while the non-overlapping parts show taxa unique to a single approach. Box plot with overlaid data points showing the percentage of taxonomic assignment identity for Arthropoda sequences using COI and V9 markers against reference databases.

Identity values for molecular assignments remain high in both markers, with COI ranging roughly 90–100% and 18SV9 clustering around 97–100%. The species table in the supplementary material Table SIII also includes four arthropod taxa labelled “Non.seq.dep.”, indicating the absence of publicly deposited sequences at the time of this study. As expected, these taxa appear only in the morphological dataset and do not occur in either COI or 18SV9 (Fig. 5).

### Meroplankton

#### Anellida

The 18SV9 region detected 19 annelid taxa, while morphology identified 3 taxa and COI 4 taxa. 18SV9 included all morphologically identified taxa and contributed 18 additional detections, whereas COI showed no overlap with the other methods. Both markers showed high assignment identity, with COI ranging 97–100% and 18SV9 concentrated around 99–100%. The species list (Table SIII**)** indicates that 18SV9 captured several larval lineages (e.g. Sabellidae, Spionidae, Oweniidae) not detected by morphology or COI, consistent with its higher sensitivity to meroplanktonic stages reported in previous work (Di Capua *et al.,* 2024) (Fig. S2).

#### Bryozoa

Bryozoans were detected exclusively by the 18SV9 marker. 18SV9 recovered three taxa two of which were identified at species level (*Bugula neritina* (Linneaus 1758) and *Parasmittina sp.*). Assignment identity for the two 18SV9 OTUs was high, with values close to 100% (Fig. S3).

#### Chordata

For Chordata, the three approaches detected only a limited set of teleost taxa, but each method captured a different subset. COI identified *Awaous commersoni* (Schneider, 1801) and *Solea solea* (Linneaus 1758); whereas 18SV9 detected *Alosa alosa* (Linneaus 1758), *Sprattus sprattus* (Linneaus 1758), and *Callionymus lyra* Linneaus 1758. Morphology recorded chordates only as Teleostei, without species-level resolution. This is expected, as fish eggs and early larval stages, common components of the mesozooplankton, cannot be reliably identified morphologically (Fig. S4).

#### Echinodermata

For Echinodermata, the three methods show moderate but uneven detection. 18SV9 detects the highest number of taxa (7 taxa), followed by morphology (5 taxa) and COI (2 taxa). The Venn diagram indicates that most detections are method-specific, with limited overlap among the three approaches **(**Fig. S5).

#### Mollusca

For Mollusca, the three methods show markedly different detection capacities: 18SV9 detects 21 taxa, COI recovers only 6 taxa, and morphology only 2 taxa. Mollusca are almost exclusively present in the mesozooplankton at the larval stage, forming an important component of meroplankton. As a consequence, the majority of molluscs are not identified under the stereomicroscope, while molecular approaches are more efficient. The Venn diagram confirms that most detections are method-specific, with COI contributing to the smallest and least overlapping subset. At the taxonomic level, 18SV9 amplicon analysis recovers a broad representation of bivalves, identifying taxa across Hiatellidae, Pharidae [*Ensis siliqua* (Linneaus 1758)], Tellinidae, Lasaeidae (*Kurtiella*), Gastrochaenidae, Limidae (*Limaria*), Mytilidae (*Musculus*, *Mytilus*), and Mactridae [*Spisula subtruncata* (da Costa, 1778)]. COI, however, fails to detect a single bivalve, even though several bivalve taxa are present. The few taxa detected by COI belong exclusively to non-bivalve molluscs (Fig. S6).

#### Nemertea

Detection of Nemertea was extremely limited across methods: 18SV9 detects 5 taxa, whereas morphology detects 1, and COI detects none. The Venn diagram confirms this pattern, as all detections are restricted to morphology and 18SV9, with no overlap with COI. Overall, 18SV9 is the only method providing meaningful detection of Nemertea, while COI fails entirely and morphology recovers only at the class level. (Fig. S7).

#### Phoronida

Detection of Phoronida was extremely limited across all methods: 18SV9 detected only the genus *Phoronis* and unassigned Phoronida as morphology (this probably as consequence of the only presence of larval phase in the samples), while COI detects none (Fig. S8).

#### Platyhelminthes

Platyhelminthes were recovered by both 18SV9 and morphological analysis, but only at high taxonomic level, with identification limited to the phylum (Fig. S9).

### Gelatinous plankton

#### Chaetognatha

Chaetognaths were detected by all three approaches with different levels of coverage. Morphology identified one taxon, the 18SV9 region detected four taxa, and COI detected two taxa. As expected, morphology is inherently dependent on the analyst’s taxonomic expertise; when a group is difficult to resolve, specimens may be conservatively reported at higher ranks (in this case), reducing apparent taxonomic resolution relative to molecular markers. The Venn diagram shows limited overlap: one taxon was shared by morphology and V9, and no taxa were common to all three methods. Most detections remained exclusive to a single approach. Specifically, COI detected *Parasaggita setosa* (J. Müller, 1847) and *Pseudosagitta lyra* (Krohn, 1853), whereas 18SV9 detected *Parasagitta elegans* (Verrill, 1873) (Fig. S10).

#### Chordata

Appendicularians were detected mostly by 18SV9, which recovered both *Fritillaria* and *Oikopleura* taxa. Morphological identifications remained at genus level, while the complete absence of Appendicularia in the COI dataset is consistent with well-documented limitations of this marker for the group (Bucklin *et al*., 2018; Albaina *et al.,* 2024). In particular, the standard COI primers commonly used in metabarcoding (Folmer *et al.,* 1994; Geller *et al.,* 2013) have proven unreliable for amplifying larvacean COI, further reducing detectability (Fig. S4).

Ascidiacea and Thaliacea were identified only by the morphological approach.

#### Cnidaria

The three methods show clear differences in the detection of cnidarian taxa. 18SV9 detects 20 taxa, far more than morphology (7 taxa) and COI (3 taxa). The Venn diagram shows that most taxa are uniquely recovered by 18SV9, while morphology and COI capture only small and partially overlapping subsets. Despite this difference in richness, both molecular markers show high identity values, indicating that the limitation of COI lies in detection, not in assignment accuracy. In our dataset, this limitation is further exacerbated by the lack of true universality of the chosen COI primer set across Cnidaria, by mitochondrial peculiarities such as duplicated COI regions and the presence of NUMTs (Bucklin *et al.,* 2019), and by the relatively long length of the 313 bp COI fragment used here, which is suboptimal for extensively degraded DNA from formalin-preserved material. In contrast, the shorter length of the 18SV9 fragment likely explains its consistently higher sensitivity (Fig. S11).

#### Ctenophora

For Ctenophora, detection was low and showed little overlap among methods: COI recovered three taxa, whereas both 18SV9 and morphology recovered only two. At the species level, the only agreement between molecular and morphological identification was *Mnemiopsis leidyi* A. Agassiz, 1865, which was detected by COI and morphology. *Cestum veneris* Lesueur, 1813 was also recovered in the COI dataset, but only as *cf.*, indicating uncertainty in the assignment (Fig. S12).

## DISCUSSION

Most current knowledge of mesozooplankton variability in the Gulf of Trieste builds on early investigations by the Austrian zoologist A. Steuer (1902a, 1902b) and on a long-term monitoring time series initiated in the 1970s at a station located 200 m offshore of Miramare Castle (45°42′2.99″ N; 13°42′36.00″ E), at the boundary of the Miramare Marine Protected Area. The station joined the LTER network in 2006 (LTER Gulf of Trieste site: https://deims.org/96969205-cfdf-41d8-979f-ff881ea8dc8b*)*. This dataset represents one of the longest-running zooplankton collections in the Mediterranean Sea, spanning more than three decades and documenting intra-annual and interannual variability (Kamburska *et al*., 2009; Giani *et al.,* 2012; Pierson *et al.,* 2021). As for many historical plankton collections worldwide, the series in the Gulf of Trieste was established before molecular approaches became part of routine taxonomic practice. The majority of these samples were collected in an era when species identification relied exclusively on morphology, long before DNA-based taxonomy and high-throughput sequencing became integrated tools for biodiversity assessment. As a result, its samples constitute a unique historical asset whose preservation is essential. Because molecular analyses require destructive subsampling, we chose not to process material from the LTER Gulf of Trieste time-series archive. Instead, to safeguard this irreplaceable collection, we optimized and tested our workflow on samples collected at a nearby site, less than 400 m away: the meteo-oceanographic buoy MIRAMARE (MAMBO-1; 45°41′51.7″ N, 13°42′29.9″ E), which is part of the ICOS Integrated Carbon Observation System network.

Formalin-preserved material can yield usable DNA, but recovery stays difficult and inconsistent, shaped by preservation time, temperature and tissue type, with fragmentation and damage-driven artifacts a recurring problem (Bucklin and Allen, 2004; Ruane and Austin, 2017; Hou *et al.,* 2021; O’Connell *et al*., 2021; Straube *et al.,* 2021; Hahn *et al*., 2022; Appleyard *et al.,* 2022; Guo *et al.,* 2022). Bulk, community-level metabarcoding of formalin-preserved zooplankton remains largely untested, and to our knowledge it remains uncommon to target a COI fragment as long as 313 bp in this context. Among the molecular alterations caused by formalin, cross-linking between nucleic acids and proteins is often considered a critical obstacle. However, cross-links are not inherently destructive and can be reversed under alkaline conditions (National Research Council (2006)). Gould *et al*., 2021 showed that alkaline lysis alone can effectively break formalin-induced cross-links and enable high-quality DNA recovery. Building on this chemical principle, following Hahn *et al.* (2022) we preferred hot alkaline lysis, combining alkaline conditions with elevated temperatures (120°C) to further enhance cross-link reversal. In our study, we adopted the same approach but applied a milder thermal regime, using 98°C rather than 120°C, to accommodate the condition of our samples while still promoting cross-link disruption. To complement this treatment, we incorporated proteinase K digestion. Although standard protocols recommend incubation at 55°C, several studies have shown that increasing the digestion temperature can improve the efficiency of the treatment without inactivating the enzyme (Shiozaki *et al.,* 2021; Straube *et al.,* 2021). Consistent with previous reports using proteinase K based extractions on formalin-preserved tissues (Ruane and Austin, 2017; O’Connell *et al.,* 2021), this combined strategy yielded sufficient gDNA for downstream sequencing. As expected for samples subjected to alkaline extraction and high-temperature treatment, the recovered gDNA from our samples was largely denatured. Then we opted not to pursue phenol-chloroform extraction (often used in these cases) and instead used a commercial kit to maximize workflow standardization, ensure consistency across all samples, and maintain a simpler, more streamlined protocol. While some studies recommend deep sequencing or even whole-genome approaches to mitigate artifacts associated with damaged templates (Burrell *et al.,* 2015), WGS is not easily applicable to multispecies mixtures like zooplankton bulk and would require extensive sequencing depth and demanding bioinformatic reconstruction. Fully aware of these limitations, we nevertheless chose to pursue an amplicon-based workflow as a pragmatic strategy to recover meaningful taxonomic information from formalin-preserved material. Accordingly, we adopted a multi-primer approach, which has become standard practice in metabarcoding studies to mitigate marker-specific biases and maximize taxonomic coverage. All three primer sets required unusually high PCR cycle numbers to produce amplicons, however that was a predictable outcome given the severe degradation caused by formalin. The 18SV9 region represented the safest option, as its short amplicon offered a higher likelihood of being successfully amplified from extensively fragmented templates. In contrast, the use of COI involved a calculated risk: the fragment is substantially longer and therefore more sensitive to formalin-induced fragmentation. However, COI offers far greater species-level resolution and can mitigate primer-specific detection biases among metazoan groups (Bucklin *et al.,* 2019; Stefanni *et al.,* 2018; Schroeder *et al*., 2021). Including this marker allowed us to test whether such resolution could still be achieved despite the compromised nature of the gDNA. As expected, COI required several optimization attempts to keep the cycle number below 55, including adjustments in annealing temperature. The 18SV4 marker was incorporated for similar reasons, as several studies suggest that 18SV4 may outperform 18SV9 in taxonomic resolution (Blanco-Bercial, 2020; Questel *et al.,* 2021). Nevertheless, it remained the most challenging of the three markers due to its relatively long amplicon size. It’s noticeable that 18SV4 amplified under similar cycle numbers to 18SV9, yet, as shown by our results, this did not translate into effective recovery of metazoan taxa. Instead, the 18SV4 reactions predominantly amplified non-metazoan DNA, reflecting the strong taxonomic bias inherent to this primer set under formalin- preserved conditions.

### Informatic pipeline

Given the complexity of our samples, we adapted the bioinformatic pipeline to better accommodate the types of damage introduced by formalin. After merging paired-end reads, we used DADA2 as a first filtering step because it removes a substantial fraction of the sequencing noise that characterizes these datasets. We initially set the maximum expected error at 8, following Shiozaki *et al.* (2021), and then lowered this value gradually until we retained about 65% of reads per sample. Formalin modifies DNA through several chemical reactions, but whether these changes accumulate in a random or structured way is still unclear. Most available evidence comes from FFPE (formalin fixed paraffin embedded) human tissues, which remain in formalin for short periods. Some studies describe the resulting DNA as a readable but damaged template in which alterations appear broadly random (Quach *et al.,* 2004), and others emphasize the extent of fragmentation and its impact on sequencing (Hykin *et al.,* 2015). In contrast, Guo *et al.* (2022) found substitution patterns, such as from thymine to cytosine changes, that do not distribute uniformly along the genome, pointing to non-random damage shaped by specific biochemical or batch effects. None of these studies, however, reflect the conditions of our material. Our zooplankton bulk samples remained seven years in formalin at room temperature before being transferred to ethanol. Ethanol introduces its own chemical modifications, including base adducts, and is widely used in museums as a safer long-term preservative, even though it further compromises DNA integrity. The combination of prolonged formalin exposure, fragmentation and ethanol-induced modifications makes the structure of damage in our samples difficult to predict. Because of this uncertainty, denoising alone was not enough. We added a clustering step to obtain OTUs that better represented the underlying community. DGC, the default in QIIME2, assumes that sequence differences arise from biological divergence and random sequencing errors. This assumption becomes unreliable when chemical damage may accumulate non-uniformly. For this reason, we exported the sequences and applied AGC in VSEARCH. By clustering around the most abundant templates, AGC reduces the effect of rare variants produced by prolonged chemical degradation and yields centroids that more reliably reflect the biological signal.

### Fungal persistence and over-representation under formalin fixation

Despite using primers commonly applied in metazoan studies, a substantial fraction of the recovered sequences belonged to non-metazoan taxa. This pattern was most evident for the 18SV4 marker, in which fungal reads dominated several months and in some cases accounted for nearly the entire dataset. The TAReukFWD1-TAReukREV3 primers are designed to amplify a broad range of eukaryotes, including Fungi, yet the extent to which fungal templates displaced metazoan DNA in our zooplankton samples remains unexpected. Evidence from preserved material suggests that formalin may not fully prevent fungal persistence. Kwizera and Naluzze (2024), in a review of eight studies published between 2002 and 2022, reported multiple fungal isolates persisting, and in some cases increasing, in cadavers stored in 5–14% formalin. These studies described fungal occurrence on cadaver surfaces, in storage pools, and even in the formalin itself (Herlina *et al*., 2022; Yaragalla and Rajput, 2017), with air, water, and the cadavers themselves identified as recurrent sources. Most of these observations derive from tropical or subtropical environments, where high humidity likely promotes fungal colonization; in some cases, cadavers also showed visible mold. By contrast, our samples were collected and stored at room temperature and in dark conditions and showed no macroscopic evidence of fungal growth. The composition of the 18SV4 dataset is consistent with this literature. The relatively long 18SV4 amplicon, together with the severe fragmentation of metazoan DNA in formalin-fixed material, likely favored amplification of more intact, multi-copy fungal rDNA templates. The strong fungal signal in 18SV4 may therefore reflect a combination of fungal DNA already present at sampling and the persistence, or possible relative enrichment during storage, of formalin-tolerant fungal taxa.

### Constraints imposed by formalin-induced DNA fragmentation

Because formalin-induced DNA degradation disrupts quantitative relationships among taxa, ecological metrics (alpha and beta diversity, community dissimilarity, ordination) cannot be interpreted reliably. We therefore focused on evaluating marker performance rather than ecological structure, comparing the taxonomic breadth, resolution and confidence delivered by COI and 18SV9 across major metazoan groups. This comparison also requires caution. Long-term formalin exposure produces highly fragmented DNA, and fragment length becomes a primary driver of amplification success. COI targets a much longer region than 18SV9, and this reduces the number of fragments that remain long enough to serve as PCR templates. Under these conditions, primer performance cannot be assessed in a conventional sense. Even well-designed COI primers will fail when template fragments fall below the minimum length required for amplification. In contrast, the shorter 18SV9 region has a higher probability of encountering intact fragments during early PCR cycles, which likely explains its broader taxonomic coverage regardless of primer design. Several studies have shown that zooplankton metabarcoding presents substantial challenges even under optimal preservation conditions. For example, Bucklin *et al.* (2021) describe how taxonomic groups differ widely in primer compatibility, mitochondrial structure, and barcode availability, resulting in uneven detection across markers. In other words, achieving full zooplankton coverage is difficult *a priori*, regardless of sample quality. When formalin is added on top of this inherent complexity, the limitations become even more pronounced. Our dataset reflects this scenario clearly. Long-term formalin exposure reduces DNA fragments to extremely short sizes, which disproportionately affects longer markers such as COI. This helps explain why COI shows patchy and taxon-biased detection in our samples, while 18SV9, because of its much shorter amplicon, recovers a broader range of phyla. Some patterns, such as the complete absence of bivalves in the COI dataset or the exclusive recovery of appendicularians in 18SV9, mirror what the literature describes: each marker retains intrinsic blind spots, and formalin simply magnifies these biases. Under such constraints, it is unrealistic to expect any single marker to represent the full zooplankton assemblage, and even the combination of COI and 18SV9 cannot guarantee exhaustive taxonomic coverage. This does not reduce the value of the dataset: it still provides biologically meaningful signals, particularly for non-indigenous species. Our samples (collected in 2017) contain multiple taxa already recognized as established or emerging invaders in the region. The calanoid copepod *P. marinus* was first reported in Mediterranean Sea in the *northern* Adriatic near Rimini (Italy) in 2007 and in the harbour of Monfalcone (Italy) in 2009 (de Olazabal and Tirelli, 2011), and has since become a characteristic component of the plankton community in the Gulf of Trieste (Uttieri *et al.,* 2020, 2023; Tirelli *et al.,* 2024). In addition, *M. leidyi*, first recorded in the Gulf of Trieste in 2005 and observed again during northern Adriatic blooms in 2016 (Malej *et al.,* 2017; Putelli *et al.,* 2025; Schroeder *et al.* 2023), was also detected in our dataset using the COI marker. The 18SV9 dataset instead includes the amphipods *Grandiderella japonica* and *J. slatteryi*, both recognized as non-indigenous or cryptogenic in the Mediterranean. *G. japonica* was reported for the first time in the Mediterranean Sea only recently, from northern Italian lagoons (Munari *et al.,* 2016), while *J. slatteryi* was confirmed from Italian coastal waters in 2018 (Bonifazi *et al.,* 2018). Our 2017 records therefore fall within the early phase of their documented regional spread. These observations highlight a practical aspect of routine zooplankton monitoring: few institutes have in-house experts capable of identifying all taxonomic groups. Metabarcoding can therefore act as an effective first screening step, helping to flag unexpected or biogeographically unusual taxa and indicating when specialist input is required. Even when formalin constrains quantitative interpretation, DNA-based detection can guide targeted morphological verification (Holland *et al.,* 2025) and contribute to reconstructing the local invasion history and broader biological assemblage dynamics of a site.

## CONCLUSIONS

This study is, to our knowledge, among the few community-level assessments of metabarcoding performance on bulk mesozooplankton preserved in formalin, and one of the few to combine this with a COI fragment long enough to support a genuine multi-primer approach. Building on previous work using formalin-preserved samples across different methods, tissues, and study aims, we show that a multi-marker strategy can still recover meaningful taxonomic information from a bulk mesozooplankton assemblage after several years of formalin storage. Formalin-preserved material remains widely available in historical collections but is rarely exploited for molecular work. Nevertheless, the combination of markers allowed us to recover a substantial portion of the metazoan assemblage and to identify taxa of ecological relevance, including non-indigenous species whose early presence may otherwise have gone unnoticed. Although important uncertainties remain, such as the role of fungal persistence and the unpredictable behavior of heavily degraded templates, our findings show that formalin-preserved samples can still provide valuable biological information when approached with appropriate caution. This work underscores the need for improved protocols and marker design tailored to degraded DNA, and highlights the potential of historical plankton archives for reconstructing past community dynamics and informing future monitoring strategies.

## Supplementary Material

Supplementary_material_fbag066

## Data Availability

The datasets presented in this study are available under the DOI: 10.5281/zenodo.20137158
