# Supplementary material for: Seven years in formalin: the burden of metabarcoding mesozooplankton preserved in buffered formalin from the northern Adriatic Sea
Source: J Plankton Res. 2026 Aug 21;48(5):fbag066. doi: 10.1093/plankt/fbag066 (PMC13496245; doi:10.1093/plankt/fbag066)
Supplement: Supplementary file 1 [file supplementary_material_fbag066.zip › Fig.S10_Chaetognatha_figure_taxa_fbag066.pdf]

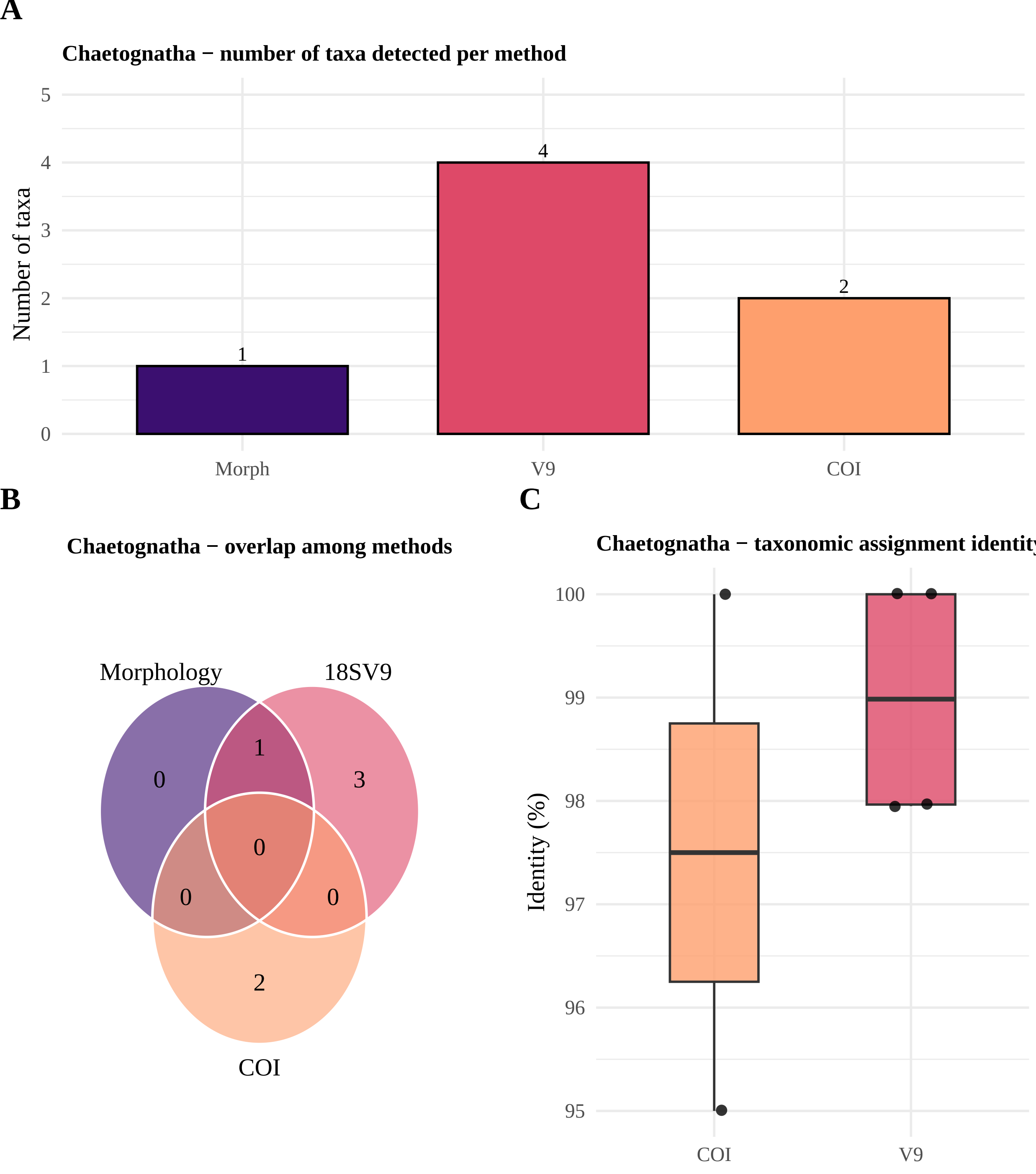
A
Chaetognatha − number of taxa detected per method
Number of taxa
5
4
3
2
1
0
1
4
2
Morph
V9
COI
B
Chaetognatha − overlap among methods
Morphology
18SV9
COI
0
1
3
0
0
0
2
C
Chaetognatha − taxonomic assignment identity
Identity (%)
100
99
98
97
96
95
COI
V9
