# Supplementary material for: Seven years in formalin: the burden of metabarcoding mesozooplankton preserved in buffered formalin from the northern Adriatic Sea
Source: J Plankton Res. 2026 Aug 21;48(5):fbag066. doi: 10.1093/plankt/fbag066 (PMC13496245; doi:10.1093/plankt/fbag066)
Supplement: Supplementary file 1 [file supplementary_material_fbag066.zip › Fig.S1_fbag066.pdf]

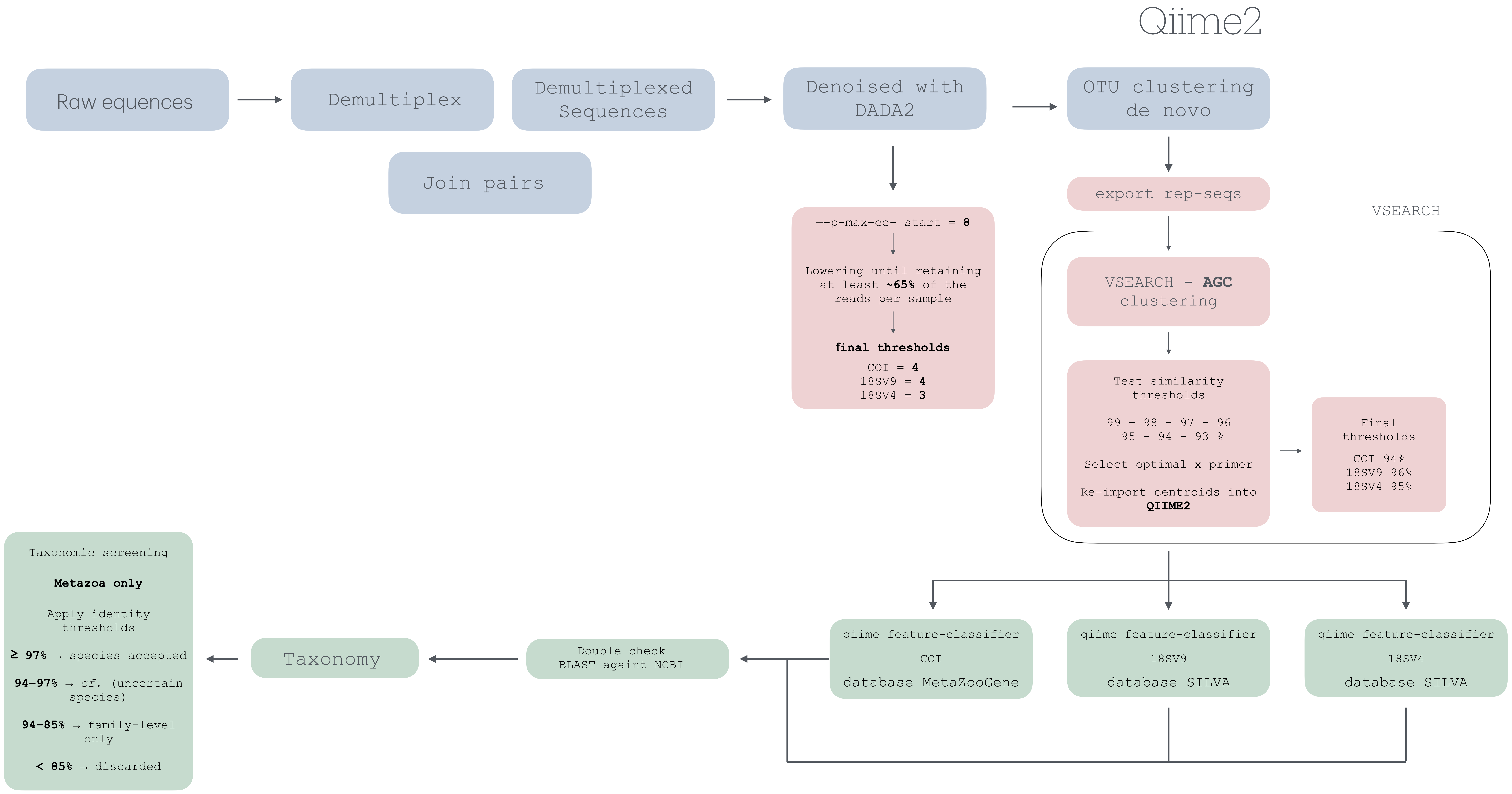

Qiime2
Raw equences
Demultiplex
Demultiplexed Sequences
Join pairs
Denoised with DADA2
OTU clustering de novo
--p-max-ee- start = **8**
Lowering until retaining at least **~65%** of the reads per sample
final thresholds
COI = **4**
18SV9 = **4**
18SV4 = **3**
export rep-seqs
VSEARCH
VSEARCH - **AGC** clustering
Test similarity thresholds
99 - 98 - 97 - 96
95 - 94 - 93 %
Select optimal x primer
Re-import centroids into **QIIME2**
Final thresholds
COI 94%
18SV9 96%
18SV4 95%
qiime feature-classifier
COI
database MetaZooGene
qiime feature-classifier
18SV9
database SILVA
qiime feature-classifier
18SV4
database SILVA
Double check
BLAST againt NCBI
Taxonomy
Taxonomic screening
Metazoa only
Apply identity thresholds
≥ 97% → species accepted
94–97% → *cf.* (uncertain species)
94–85% → family-level only
< 85% → discarded
