# Supplementary material for: Seven years in formalin: the burden of metabarcoding mesozooplankton preserved in buffered formalin from the northern Adriatic Sea
Source: J Plankton Res. 2026 Aug 21;48(5):fbag066. doi: 10.1093/plankt/fbag066 (PMC13496245; doi:10.1093/plankt/fbag066)
Supplement: Supplementary file 1 [file supplementary_material_fbag066.zip › Fig.S3_Bryozoa_figure_taxa_fbag066.pdf]

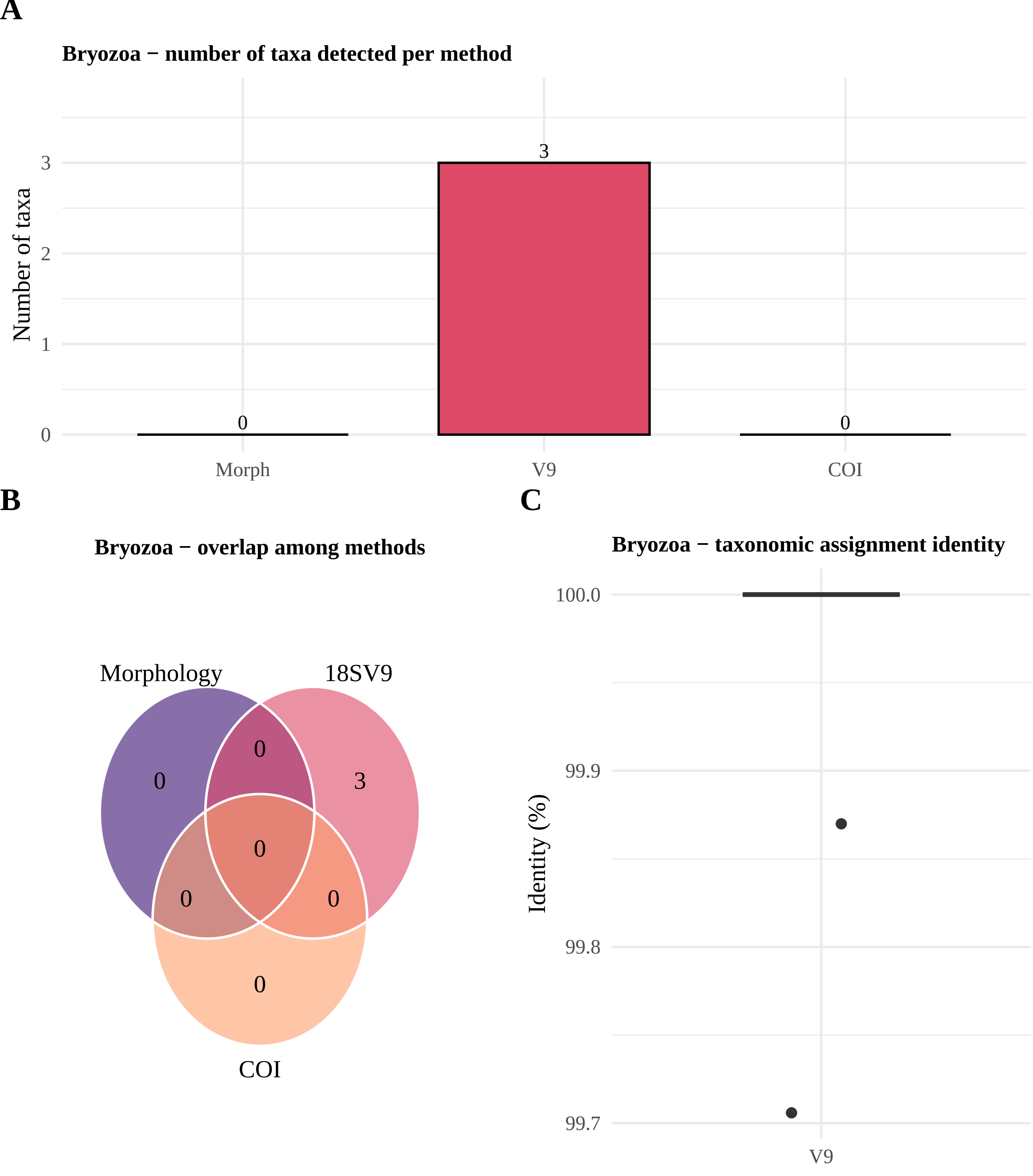
A
Bryozoa − number of taxa detected per method
Number of taxa
0
1
2
3
3
0
0
Morph
V9
COI
B
Bryozoa − overlap among methods
Morphology
18SV9
0
0
3
0
0
0
0
COI
C
Bryozoa − taxonomic assignment identity
Identity (%)
100.0
99.9
99.8
99.7
V9
