# Supplementary material for: Seven years in formalin: the burden of metabarcoding mesozooplankton preserved in buffered formalin from the northern Adriatic Sea
Source: J Plankton Res. 2026 Aug 21;48(5):fbag066. doi: 10.1093/plankt/fbag066 (PMC13496245; doi:10.1093/plankt/fbag066)
Supplement: Supplementary file 1 [file supplementary_material_fbag066.zip › Fig.S4_Chordata_figure_taxa_fbag066.pdf]

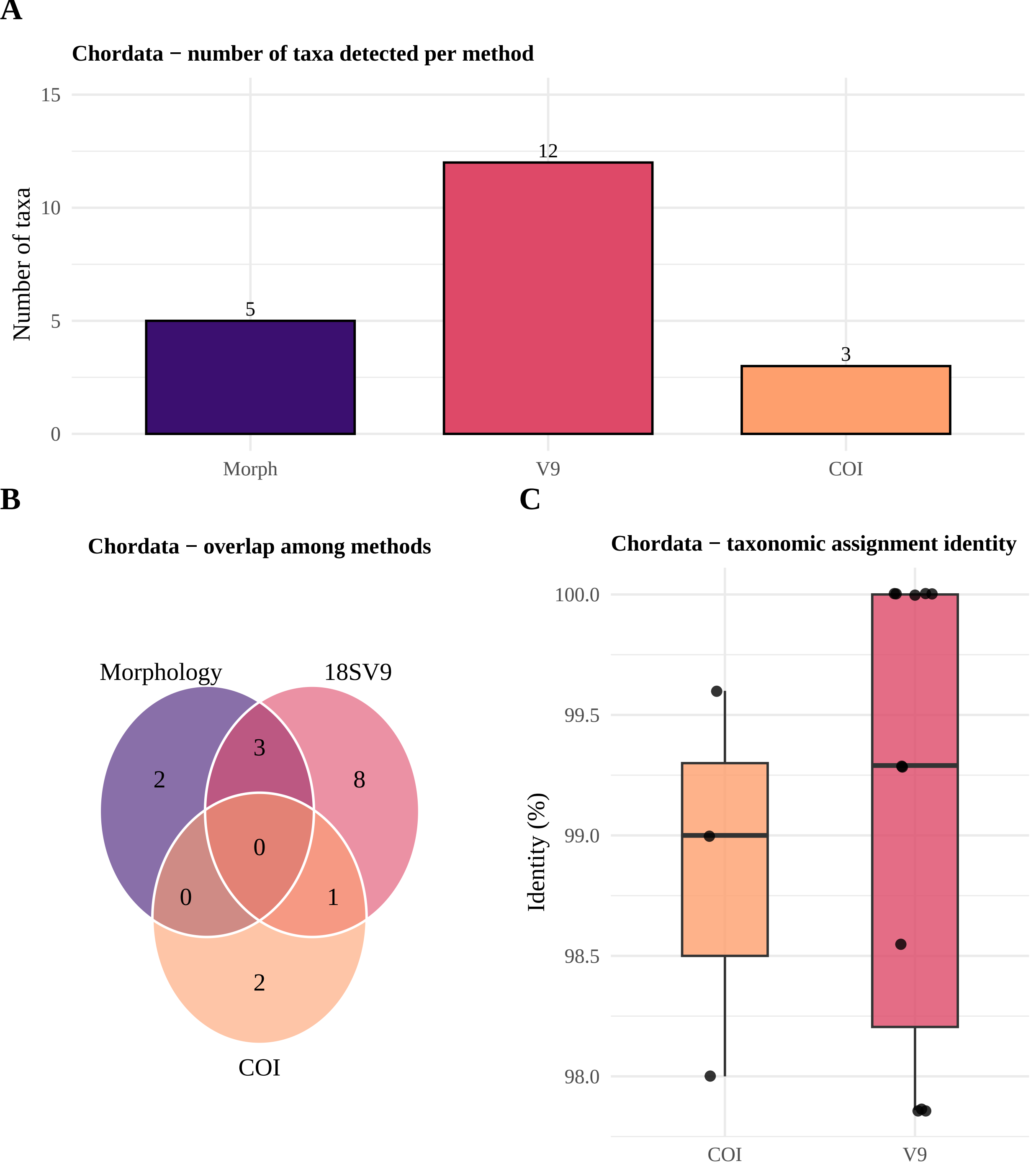
A
Chordata − number of taxa detected per method
Number of taxa
15
10
5
0
12
5
3
Morph
V9
COI
B
Chordata − overlap among methods
Morphology
18SV9
COI
2
3
8
0
0
1
2
C
Chordata − taxonomic assignment identity
Identity (%)
100.0
99.5
99.0
98.5
98.0
COI
V9
