# Supplementary material for: Seven years in formalin: the burden of metabarcoding mesozooplankton preserved in buffered formalin from the northern Adriatic Sea
Source: J Plankton Res. 2026 Aug 21;48(5):fbag066. doi: 10.1093/plankt/fbag066 (PMC13496245; doi:10.1093/plankt/fbag066)
Supplement: Supplementary file 1 [file supplementary_material_fbag066.zip › Fig.S5_Echinodermata_figure_taxa_fbag066.pdf]

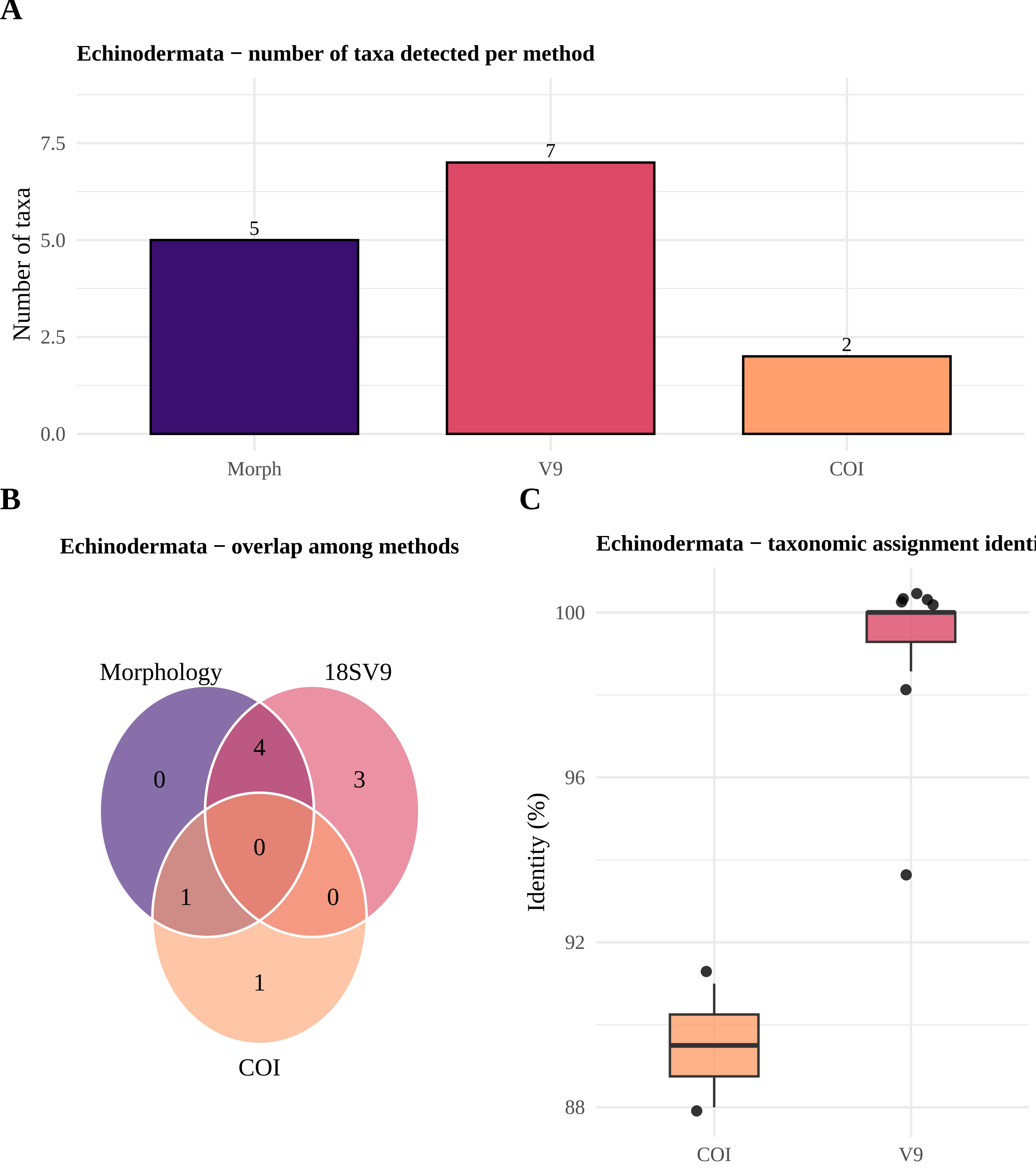

A
Echinodermata − number of taxa detected per method
Number of taxa
0.0
2.5
5.0
7.5
5
7
2
Morph
V9
COI
B
Echinodermata − overlap among methods
Morphology
18SV9
COI
0
4
3
0
1
0
1
C
Echinodermata − taxonomic assignment identi
Identity (%)
100
96
92
88
COI
V9
