# Supplementary material for: Seven years in formalin: the burden of metabarcoding mesozooplankton preserved in buffered formalin from the northern Adriatic Sea
Source: J Plankton Res. 2026 Aug 21;48(5):fbag066. doi: 10.1093/plankt/fbag066 (PMC13496245; doi:10.1093/plankt/fbag066)
Supplement: Supplementary file 1 [file supplementary_material_fbag066.zip › Fig.S7_Nemertea_figure_taxa_fbag066.pdf]

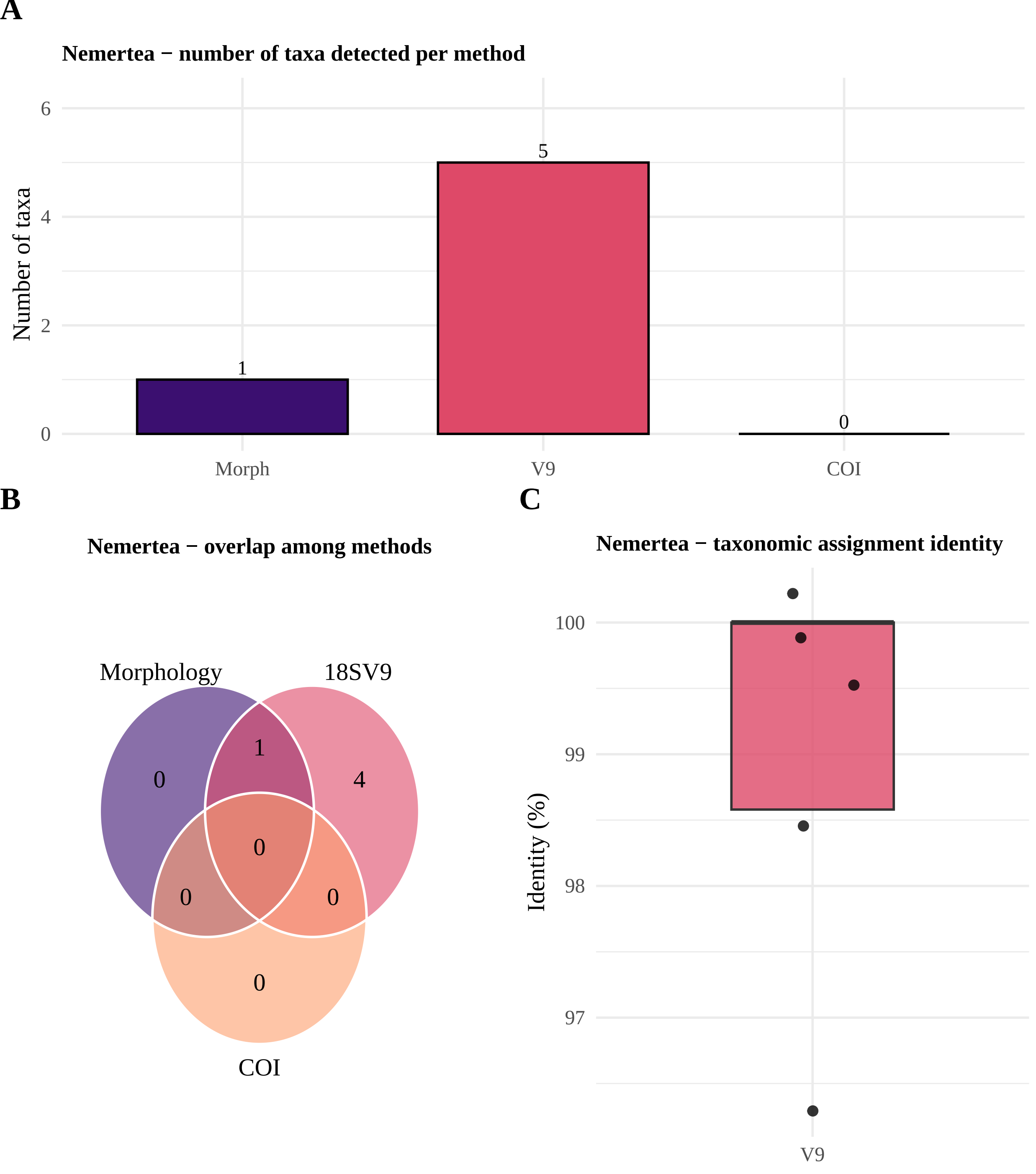
A
Nemertea − number of taxa detected per method
Number of taxa
6
4
2
0
5
1
0
Morph
V9
COI
B
Nemertea − overlap among methods
Morphology
18SV9
COI
0
1
4
0
0
0
0
C
Nemertea − taxonomic assignment identity
Identity (%)
100
99
98
97
V9
