# Supplementary material for: Seven years in formalin: the burden of metabarcoding mesozooplankton preserved in buffered formalin from the northern Adriatic Sea
Source: J Plankton Res. 2026 Aug 21;48(5):fbag066. doi: 10.1093/plankt/fbag066 (PMC13496245; doi:10.1093/plankt/fbag066)
Supplement: Supplementary file 1 [file supplementary_material_fbag066.zip › Fig.S8_Phoronida_figure_taxa_fbag066.pdf]

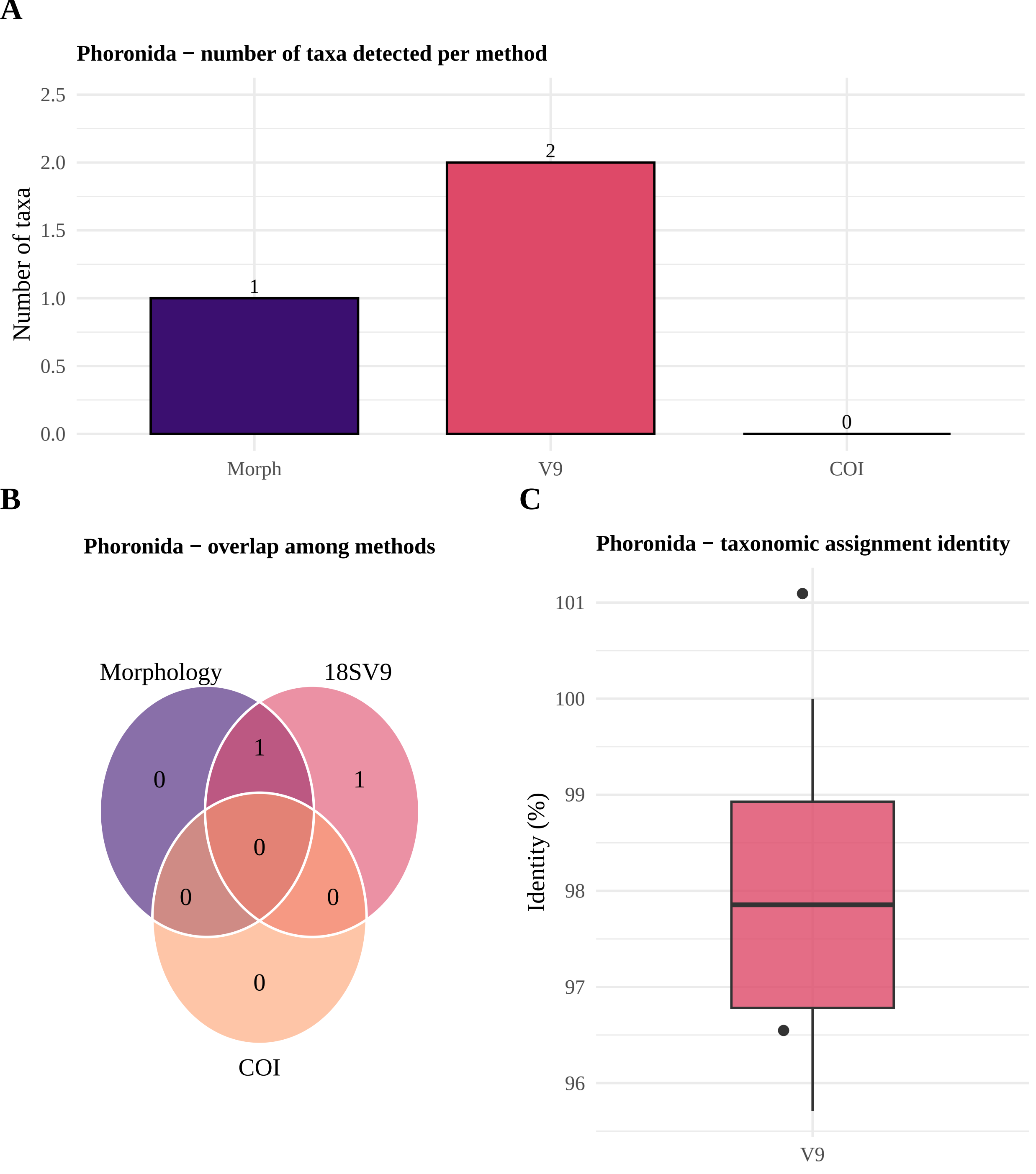

A
Phoronida − number of taxa detected per method
Number of taxa
0.0
0.5
1.0
1.5
2.0
2.5
1
2
0
Morph
V9
COI
B
Phoronida − overlap among methods
Morphology
18SV9
COI
0
1
1
0
0
0
0
C
Phoronida − taxonomic assignment identity
Identity (%)
96
97
98
99
100
101
V9
