# Supplementary material for: Seven years in formalin: the burden of metabarcoding mesozooplankton preserved in buffered formalin from the northern Adriatic Sea
Source: J Plankton Res. 2026 Aug 21;48(5):fbag066. doi: 10.1093/plankt/fbag066 (PMC13496245; doi:10.1093/plankt/fbag066)
Supplement: Supplementary file 1 [file supplementary_material_fbag066.zip › Fig.S9_Platyhelminthes_figure_taxa_fbag066.pdf]

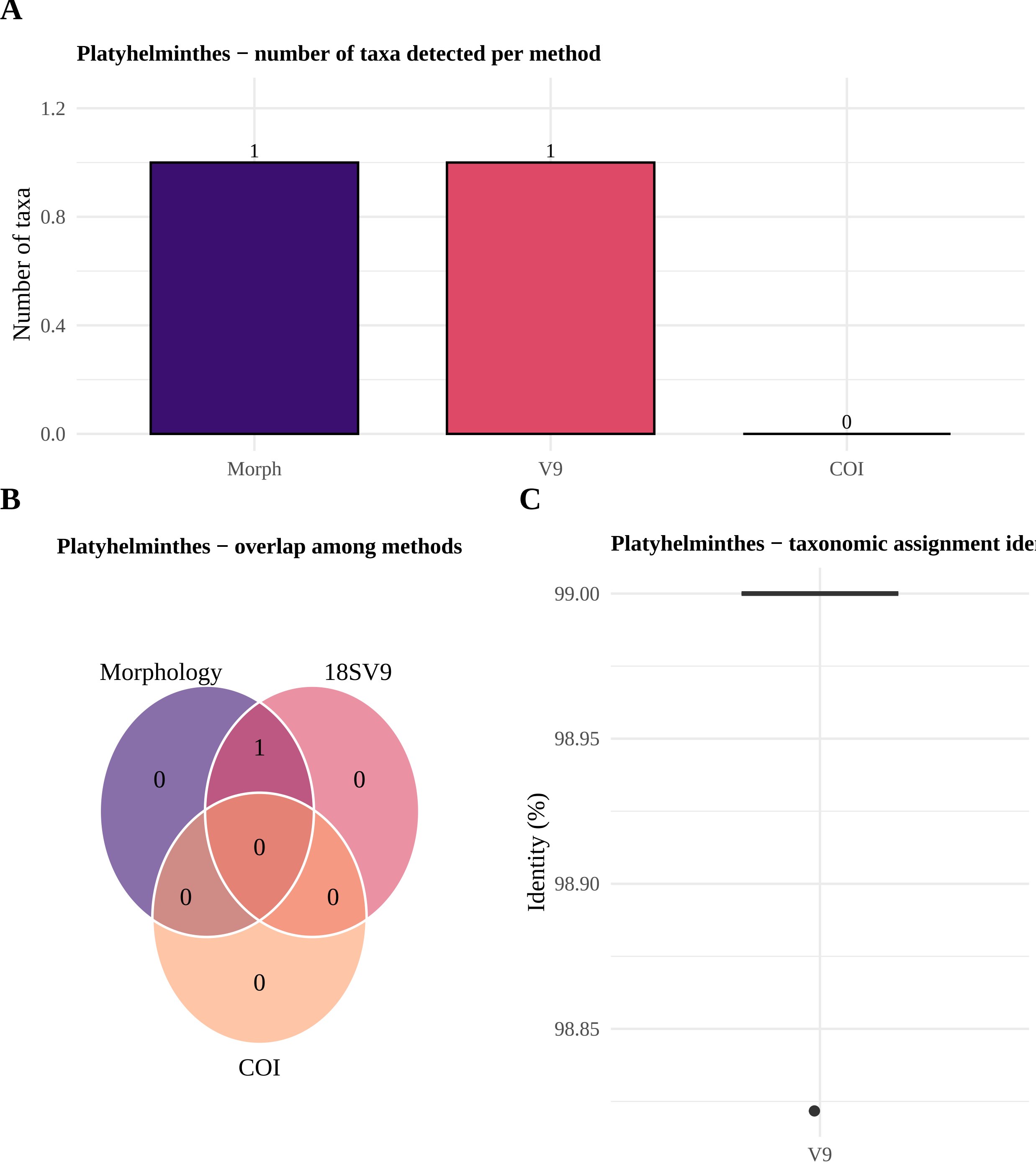
A
Platyhelminthes − number of taxa detected per method
Number of taxa
1.2
0.8
0.4
0.0
1
1
0
Morph
V9
COI
B
Platyhelminthes − overlap among methods
Morphology
18SV9
COI
0
1
0
0
0
0
0
C
Platyhelminthes − taxonomic assignment ide
Identity (%)
99.00
98.95
98.90
98.85
V9
