# Supplementary material for: Seven years in formalin: the burden of metabarcoding mesozooplankton preserved in buffered formalin from the northern Adriatic Sea
Source: J Plankton Res. 2026 Aug 21;48(5):fbag066. doi: 10.1093/plankt/fbag066 (PMC13496245; doi:10.1093/plankt/fbag066)
Supplement: Supplementary file 1 [file supplementary_material_fbag066.zip › Supplementary_Material_fbag066.docx]

**Supplementary material**

**Figures Legends**

**Fig. S1** Overview of the bioinformatics workflow used for sequence processing, de novo OTU clustering, and taxonomic assignment. Reads were processed in QIIME 2 (demultiplexing, joining, and DADA2 denoising), clustered de novo with VSEARCH (AGC), and re-imported into QIIME 2 for taxonomy assignment using marker-specific reference databases (SILVA for 18S and MetaZooGene for COI). Assignments were cross-checked with BLAST, and a final screening step retained metazoan hits and applied identity-based reporting thresholds.

Alt text: Flowchart of the bioinformatics pipeline with seven main stages, arranged as a sequence with a database branch in the middle. Raw sequences are demultiplexed, paired reads joined, then denoised with DADA2 using a starting --p-max-ee value of 8, lowered until at least ~65% of reads per sample are retained; the final denoising thresholds are 4 for COI, 4 for 18SV9, and 3 for 18SV4. The denoised sequences undergo de novo OTU clustering with VSEARCH (AGC algorithm), tested across similarity thresholds from 99% down to 93% in 1% steps, to select the optimal threshold per primer; the selected centroids are re-imported into QIIME 2 and exported as representative sequences. Taxonomic assignment then branches by primer: COI is classified against the MetaZooGene database, while 18SV9 and 18SV4 are classified against the SILVA database, each using QIIME 2's feature-classifier. The final identity thresholds selected are 94% for COI, 96% for 18SV9, and 95% for 18SV4. Assignments are double-checked with a BLAST search against NCBI, screened to retain Metazoa only, and reported according to identity: ≥97% identity is accepted at species level, 94–97% is reported as "cf." (uncertain species), 85–94% is reported at family level only, and below 85% is discarded.

**Fig. S2** Summary of Ctenophora detections across methods. (A) Number of ctenophore taxa detected by morphology, COI, and 18S V9. (B) Three-way Venn diagram showing minimal overlap; detections are mostly method-specific. (C) Assignment identity (%) for COI and 18SV9. *M. leidyi* is the only taxon detected by two methods.

Alt text: Three-panel figure for phylum Ctenophora. Panel A shows the number of taxa detected: 2 by Morphology, 2 by 18SV9, and 3 by COI. Panel B is a Venn diagram in which *M.leidyi* is the only taxon detected by more than one method, shared between COI and Morphology; all other detections are method-specific. Panel C shows taxonomic assignment identity for COI and 18SV9.

**Fig. S3** Summary of Cnidaria detections across methods. (A) Number of cnidarian taxa detected by morphology, COI, and 18SV9. (B) Three-way Venn diagram showing that most detections occur exclusively in 18SV9. (C) Assignment identity (%) for COI and 18SV9. Despite lower richness, COI assignments show high identity values.

Alt text: Three-panel figure for phylum Cnidaria. Panel A shows the number of taxa detected: 7 by Morphology, 20 by 18SV9, and 3 by COI. Panel B is a Venn diagram in which most taxa are detected exclusively by 18SV9, with Morphology and COI capturing smaller, partially overlapping subsets. Panel C shows taxonomic assignment identity for COI and 18SV9; both markers show high identity values despite COI's lower taxon count.

**Fig. S4** Summary of Chaetognata detections across methods. (A) Number of chaetognath taxa detected by morphology, COI, and 18SV9. (B) Three-way Venn diagram showing limited overlap among methods; most detections are method-specific. (C) Assignment identity (%) for COI and 18SV9. Both markers show consistently high identity values.

Alt text: Three-panel figure for phylum Chaetognatha. Panel A shows the number of taxa detected: 1 by Morphology, 4 by 18SV9, and 2 by COI. Panel B is a Venn diagram showing one taxon shared between Morphology and 18SV9, and no taxa shared among all three methods; most detections are method-specific. At species level, COI detected Parasagitta setosa and Pseudosagitta lyra; 18SV9 detected Parasagitta elegans. Panel C shows taxonomic assignment identity for COI and 18SV9, both consistently high.

**Fig. S5** Summary of Bryozoa detections across methods. (A) Number of bryozoan taxa detected by morphology, COI, and 18SV9. (B) Three-way Venn diagram showing that detections occur exclusively in 18SV9. (C) Assignment identity (%) for 18SV9. Both detected OTUs show identity values close to 100%.

Alt text: Three-panel figure for phylum Bryozoa. Panel A shows 3 taxa detected, all by 18SV9 only; Morphology and COI detected none. Panel B is a Venn diagram with all 3 taxa exclusive to the 18SV9 circle. Panel C shows taxonomic assignment identity for the two 18SV9 OTUs identified to species level, both close to 100%.

**Fig. S6** Summary of Phoronida detections across methods. (A) Number of phoronid taxa detected by morphology, COI, and 18S V9. (B) Three-way Venn diagram showing no COI detections and one detection each in morphology and 18S V9. (C) Assignment identity (%) for 18S V9. The single detection shows high identity.

Alt text: Three-panel figure for phylum Phoronida. Panel A shows 1 taxon detected by Morphology, 1 by 18SV9, and 0 by COI. Panel B is a Venn diagram showing the Morphology and 18SV9 detections as separate, non-overlapping single detections, with no COI detections. Panel C shows a high taxonomic assignment identity value for the single 18SV9 detection.

**Fig. S7** Summary of Nemertea detections across methods. (A) Number of nemertean taxa detected by morphology, COI, and 18S V9. (B) Three-way Venn diagram showing detections restricted to morphology and 18S V9. (C) Assignment identity (%) for 18S V9. All V9 detections show high identity values.

Alt text: Three-panel figure for phylum Nemertea. Panel A shows the number of taxa detected: 1 by Morphology, 5 by 18SV9, and 0 by COI. Panel B is a Venn diagram showing all detections restricted to Morphology and 18SV9, with no COI involvement. Panel C shows taxonomic assignment identity for the 18SV9 detections, all high.

**Fig. S8** Summary of Platyhelminthes across methods (A) Number of taxa detected by morphology, 18SV9, and COI. (B) Overlap among methods. Platyhelminthes were detected by both morphology and 18SV9, but not by COI. (C) Taxonomic assignment identity for the 18SV9 detection.

Alt text: Figure for phylum Platyhelminthes. Panel A shows taxa detected by Morphology and 18SV9, none by COI. Panel B shows overlap between Morphology and 18SV9 detections, with no COI involvement. Panel C shows the taxonomic assignment identity value for the single 18SV9 detection.

**Fig. S9** Summary of Mollusca detections across methods. (A) Number of molluscan taxa detected by morphology, COI, and 18SV9. (B) Three-way Venn diagram showing limited overlap; COI detects the lowest number of taxa. (C) Assignment identity (%) for COI and 18SV9. COI detects no bivalves, whereas morphology and 18S V9 recover a broad molluscan representation.

Alt text: Three-panel figure for phylum Mollusca. Panel A shows the number of taxa detected: 2 by Morphology, 21 by 18SV9, and 6 by COI. Panel B is a Venn diagram showing limited overlap among methods, with COI contributing the fewest and least overlapping taxa. 18SV9 detected bivalve taxa across Hiatellidae, Pharidae, Tellinidae, Lasaeidae, Gastrochaenidae, Limidae, Mytilidae and Mactridae; COI detected no bivalves, only non-bivalve molluscs. Panel C shows taxonomic assignment identity for COI and 18SV9.

**Fig. S10** Summary of Annelida detections across methods. (A) Number of annelid taxa detected by morphology, COI, and 18SV9. (B) Three-way Venn diagram showing unique and shared detections; COI shows no overlap with the other methods. (C) Assignment identity (%) for COI and 18SV9. The 18SV9 region detected the highest number of taxa, including all morphologically identified taxa.

Alt text: Three-panel figure for phylum Annelida. Panel A shows the number of taxa detected: 3 by Morphology, 19 by 18SV9, and 4 by COI. Panel B is a Venn diagram showing 18SV9 includes all Morphology-detected taxa, with COI showing no overlap with either of the other two methods. Panel C shows taxonomic assignment identity for COI and 18SV9, with COI ranging from about 97% to 100% and 18SV9 concentrated around 99% to 100%.

**Fig. S11** Summary of Echinodermata detections across methods. (A) Number of echinoderm taxa detected by morphology, COI, and 18SV9. (B) Three-way Venn diagram showing method-specific contributions with limited overlap. (C) Assignment identity (%) for COI and 18S V9. The 18SV9 marker detects the highest number of taxa.

Alt text: Three-panel figure for phylum Echinodermata. Panel A shows the number of taxa detected: 5 by Morphology, 7 by 18SV9, and 2 by COI. Panel B is a Venn diagram showing mostly method-specific contributions with limited overlap among the three approaches. Panel C shows taxonomic assignment identity for COI and 18SV9.

**Fig. S12** Summary of Chordata detections across methods. (A) Number of chordate taxa detected by morphology, COI, and 18S V9. (B) Three-way Venn diagram showing distinct and non-overlapping subsets among methods. (C) Assignment identity (%) for COI and 18S V9. Each method detects different teleost taxa, while morphology remains at class level.

Alt text: Three-panel figure for phylum Chordata, covering both teleost and gelatinous (appendicularian, ascidian, thaliacean) components. Panel B is a Venn diagram showing distinct, non-overlapping taxa detected by each method. At species level, COI detected Awaous commersoni and *Solea solea*; 18SV9 detected *Alosa alosa, Sprattus sprattus, Callionymus lyra*, plus Fritillaria and Oikopleura appendicularian taxa; Morphology recorded fish only at the Teleostei level, plus Ascidiacea and Thaliacea detected only by Morphology; COI detected no appendicularians. Panel C shows taxonomic assignment identity for COI and 18SV9.

**Table SI**

Relative abundance (%) of each Kingdom per month, for each genetic marker (18SV4, 18SV9, COI). Values correspond to the underlying percentages shown in Fig. 3 (bubble plot), reported here with full precision.

“–“ = not detected (relative abundance = 0%). “<0.01%” = detected, but below the rounding threshold at two decimal places.

## I.1 18SV4

| Month | Bacteria | Chromista | Fungi | Metazoa | Plantae | Protista |
| --- | --- | --- | --- | --- | --- | --- |
| January | – | – | 52.99% | 23.07% | 23.94% | – |
| February | – | – | 16.10% | 62.74% | 21.16% | – |
| March | – | – | 96.96% | 2.97% | 0.08% | – |
| April | – | – | 66.24% | 0.37% | 33.39% | – |
| May | – | – | 85.26% | 14.73% | <0.01% | <0.01% |
| June | – | – | 95.22% | 0.01% | 4.76% | <0.01% |
| July | – | – | 91.84% | 1.60% | 6.56% | <0.01% |
| August | – | – | 83.39% | 2.50% | 0.03% | 14.08% |
| September | – | – | 4.65% | 95.16% | 0.19% | <0.01% |
| October | – | – | 44.86% | 13.20% | 41.26% | 0.68% |
| November | – | – | 92.41% | 2.58% | 5.00% | <0.01% |
| December | – | – | 30.93% | 68.71% | 0.36% | <0.01% |

## I.2 18SV9

| Month | Bacteria | Chromista | Fungi | Metazoa | Plantae | Protista |
| --- | --- | --- | --- | --- | --- | --- |
| January | – | 0.09% | 0.71% | 98.67% | 0.01% | 0.52% |
| February | – | 0.03% | 1.46% | 98.45% | 0.01% | 0.04% |
| March | – | 0.10% | 7.07% | 80.65% | 12.14% | 0.05% |
| April | – | 1.79% | 23.14% | 71.26% | 3.81% | – |
| May | – | 0.13% | 8.44% | 91.15% | 0.09% | 0.20% |
| June | – | 0.23% | 53.63% | 41.50% | 4.65% | – |
| July | – | – | 3.91% | 95.66% | 0.32% | 0.12% |
| August | – | 0.05% | 0.66% | 97.64% | 0.10% | 1.54% |
| September | – | – | 12.30% | 87.48% | 0.14% | 0.08% |
| October | – | – | 13.83% | 78.99% | 1.06% | 6.12% |
| November | – | 0.02% | 1.84% | 97.79% | 0.04% | 0.31% |
| December | – | – | 1.31% | 98.36% | 0.06% | 0.27% |

## I.3 COI

| Month | Bacteria | Chromista | Fungi | Metazoa | Plantae | Protista |
| --- | --- | --- | --- | --- | --- | --- |
| January | 0.01% | 0.45% | 3.99% | 95.55% | – | <0.01% |
| February | 0.01% | 0.05% | 7.55% | 92.39% | – | – |
| March | <0.01% | 1.24% | 37.36% | 60.43% | 0.97% | – |
| April | 0.54% | 76.42% | 0.13% | 22.19% | 0.70% | <0.01% |
| May | <0.01% | 8.67% | 42.71% | 48.61% | – | – |
| June | 0.10% | 86.45% | 0.27% | 13.16% | – | 0.02% |
| July | 76.58% | 9.61% | 8.84% | 4.96% | – | <0.01% |
| August | 0.01% | 0.07% | 0.03% | 99.88% | – | – |
| September | 3.72% | <0.01% | 3.51% | 92.75% | – | 0.01% |
| October | 0.03% | <0.01% | 0.39% | 99.58% | – | <0.01% |
| November | <0.01% | – | 14.09% | 85.91% | – | <0.01% |
| December | 13.42% | 8.73% | 0.04% | 59.88% | <0.01% | 17.93% |

**Table SII** Comparison of COI, 18S-V4, and 18S-V9 primer sets in terms of total OTUs,

metazoan OTUs, proportion of metazoan OTUs, total reads, metazoan reads, and proportion of metazoan reads.

| Primer | OTU total | OTU metazoa | OTU metazoa % | Reads metazoa % | Reads total | Reads  metazoa |
| --- | --- | --- | --- | --- | --- | --- |
| COI | 214 | 133 | 62.15 | 55.82 | 9295580 | 5189658 |
| V4 | 50 | 18 | 36 | 13.69 | 9265624 | 1268427 |
| V9 | 524 | 367 | 70.04 | 88.04 | 6580631 | 5793417 |

**Table SIII** List of taxa identified using COI, 18S-V4, and 18S-V9 metabarcoding, as well as morphological analysis. An “x” indicates detection by the corresponding method. Species marked with an asterisk (*) lack a reference sequence for the corresponding genetic marker in public databases.

| **Phylum** | **Class** | **Order** | **Family** | **Genus** | **Species** | **COI** | **18S-V4** | **18S-V9** | **Morphology** |
| --- | --- | --- | --- | --- | --- | --- | --- | --- | --- |
| Ctenophora | Tentaculata | Cestida | Cestidae | *Cestum* | *Cestum veneris* | x cf. |  |  |  |
|  |  | Cydippida | Cydippidae | *Pleurobrachia* |  |  |  | x |  |
|  |  | Lobata | Bolinopsidae | *Mnemiopsis* | *Mnemiopsis leidyi* | x |  |  | x |
|  |  |  | Leucotheidae | *Leucothea* |  | x |  |  |  |
|  |  |  |  |  |  |  |  | x | x |
| Cnidaria | Hexacorallia | Actiniaria |  |  |  |  |  | x |  |
|  | Hydrozoa | Anthoathecata | Bougainvilliidae | *Bougainvillia* | *Bougainvillia sp.* |  |  | x |  |
|  |  |  | Bougainvilliidae |  |  |  |  | x |  |
|  |  |  | Corymorphidae | *Corymorpha* |  |  |  | x |  |
|  |  |  | Corymorphidae |  |  |  |  | x |  |
|  |  |  | Hydractiniidae | *Podocoryna* |  | x |  | x |  |
|  |  |  | Rathkeidae | *Podocorynoides* |  |  |  | x |  |
|  |  |  |  |  |  |  |  | x | x |
|  |  | Leptothecata | Campanulariidae | *Clytia* | *Clytia gracilis* |  |  | x cf. |  |
|  |  |  |  | *Obelia* | *Obelia dichotoma* | x |  | x |  |
|  |  |  |  | *Obelia* |  |  |  | x | x |
|  |  |  | Eirenidae | *Helgicirrha* |  |  |  | x |  |
|  |  |  |  |  |  |  |  | x |  |
|  |  | Limnomedusae |  |  |  |  |  | x |  |
|  |  | Siphonophorae | Diphyidae | *Muggiaea* | *Muggiaea atlantica* |  |  | x | x |
|  |  |  |  |  | *Muggiaea kochi* |  |  |  | x* |
|  |  |  | Sphaeronectidae | *Sphaeronectes* |  | x |  |  |  |
|  |  |  |  |  |  |  |  | x | x |
|  |  |  |  |  |  |  |  | x | x |
|  | Scyphozoa | Coronatae | Nausithoidae | *Nausithoe* | *Nausithoe punctata* |  |  |  | x |
|  |  | Rhizostomeae | Rhizostomatidae | *Rhizostoma* | *Rhizostoma pulmo* |  |  | x |  |
|  |  | Semaeostomeae | Ulmaridae | *Aurelia* | *Aurelia sp.* |  |  | x |  |
|  |  |  |  |  |  |  |  | x |  |
| Bryozoa | Gymnolaemata | Cheilostomatida | Bugulidae | *Bugula* | *Bugula neritina* |  |  | x |  |
|  |  |  | Smittinidae | *Parasmittina* | *Parasmittina sp.* |  |  | x |  |
|  |  |  |  |  |  |  |  | x |  |
| Phoronida |  |  | Phoronidae | *Phoronis* |  |  |  | x |  |
|  |  |  |  |  |  |  |  | x | x |
| Nemertea | Pilidiophora | Heteronemertea | Lineidae | *Cerebratulus* | *Cerebratulus lacteus* |  |  | x cf. |  |
|  |  |  |  |  |  |  |  | x |  |
|  |  | Hubrechtiiformes | Hubrechtellidae |  |  |  |  | x |  |
|  |  |  |  |  |  |  |  | x |  |
|  |  |  |  |  |  |  |  | x | x |
| Platyhelminthes |  |  |  |  |  |  |  | x | x |
| Annelida | Polychaeta | Phyllodocida | Lopadorrhynchidae | *Maupasia* | *Maupasia gracilis* |  |  | x |  |
|  |  |  | Phyllodocidae |  |  | x |  |  |  |
|  |  |  | Tomopteridae | *Tomopteris* |  | x |  |  |  |
|  |  |  |  |  |  |  |  | x |  |
|  |  | Sipuncula | Sipunculidae | *Sipunculus* | *Sipunculus (Sipunculus) norvegicus* |  |  | x |  |
|  |  | Spionida | Spionidae | *Laonice* | *Laonice grimaldii* | x cf. |  |  |  |
|  |  |  |  | *Prionospio* | *Prionospio fallax* |  |  | x |  |
|  |  |  |  |  | *Prionospio plumosa* |  |  | x |  |
|  |  |  |  | *Pseudopolydora* | *Pseudopolydora pulchra* |  |  | x |  |
|  |  |  |  | *Scolelepis* |  |  |  | x |  |
|  |  |  |  | *Spiophanes* |  |  |  | x |  |
|  |  |  |  |  |  |  |  | x |  |
|  |  | Terebellida | Pectinariidae | *Pectinaria* |  |  |  | x |  |
|  |  |  |  |  |  |  |  | x |  |
|  |  | incertae sedis | Oweniidae | *Owenia* | *Owenia fusiformis* |  |  | x |  |
|  |  |  | Chaetopteridae | *Spiochaetopterus* |  |  |  | x |  |
|  |  |  | Magelonidae | *Magelona* |  |  |  | x | x |
|  |  |  | Polygordiidae | *Polygordius* | *Polygordius jouinae* |  |  | x |  |
|  |  |  | Polygordiidae | *Polygordius* |  |  |  | x | x |
|  |  |  | Sabellariidae | *Sabellaria* | *Sabellaria alveolata* |  |  | x |  |
|  |  |  |  |  |  | x |  | x | x |
|  |  |  |  |  |  |  |  | x |  |
| Mollusca | Bivalvia | Adapedonta | Hiatellidae | *Hiatella* |  |  |  | x |  |
|  |  |  | Pharidae | *Ensis* | *Ensis siliqua* |  |  | x |  |
|  |  |  |  | *Phaxas* | *Phaxas pellucidus* |  |  | x |  |
|  |  | Cardiida | Cardiidae | *Cerastoderma* | *Cerastoderma edule* |  |  | x |  |
|  |  |  | Tellinidae |  |  |  |  | x |  |
|  |  | Galeommatida | Lasaeidae | *Kurtiella* |  |  |  | x |  |
|  |  | Gastrochaenida | Gastrochaenidae | *Rocellaria* |  |  |  | x |  |
|  |  | Limida | Limidae | *Limaria* |  |  |  | x |  |
|  |  | Mytilida | Mytilidae | *Musculus* |  |  |  | x |  |
|  |  |  |  | *Mytilus* | *Mytilus edulis* |  |  | x |  |
|  |  |  |  | *Mytilus* |  |  |  | x |  |
|  |  | Pectinida |  |  |  |  |  | x cf. |  |
|  |  | Pterioida | Pinnidae | *Atrina* | *Atrina pectinata* |  |  | x |  |
|  |  | Venerida | Mactridae | *Spisula* | *Spisula subtruncata* |  |  | x |  |
|  |  |  |  |  |  |  |  | x | x |
|  | Gastropoda | Caenogastropoda incertae sedis | Turritellidae | *Turritella* | *Turritella communis* |  |  | x cf. |  |
|  |  |  |  | *Turritellinella* | *Turritellinella tricarinata* | x |  |  |  |
|  |  | Littorinimorpha | Atlantidae | *Atlanta* |  | x |  |  |  |
|  |  | Neogastropoda | Turridae | *Gemmuloborsonia* | *Gemmuloborsonia colorata* |  |  | x |  |
|  |  | Nudibranchia | Discodorididae | *Jorunna* | *Jorunna tomentosa* |  |  | x |  |
|  |  | Pteropoda | Cliidae | *Clio* | *Clio pyramidata* | x |  |  |  |
|  |  |  | Hyalocylidae | *Hyalocylis* | *Hyalocylis striata* | x |  |  |  |
|  |  |  | Limacinidae | *Heliconoides* | *Limacina inflata* | x cf. |  |  |  |
|  |  |  | Hermaeidae | *Aplysiopsis* | *Aplysiopsis minor* |  |  | x |  |
|  |  |  | Planaxidae | *Planaxis* | *Planaxis sulcatus* |  |  | x |  |
|  |  |  |  |  |  |  |  | x | x |
| Chaetognatha | Sagittoidea | Aphragmophora | Sagittidae | *Parasagitta* | *Parasagitta elegans* |  |  | x |  |
|  |  |  |  |  | *Parasagitta setosa* | x cf. |  |  |  |
|  |  |  |  | *Pseudosagitta* | *Pseudosagitta lyra* | x |  |  |  |
|  |  |  |  | *Sagitta* |  |  |  | x | x |
|  |  |  | Sagittidae |  |  |  |  | x |  |
|  |  |  |  |  |  |  |  | x |  |
|  |  |  | Sagittidae | *Parasagitta* | *Parasagitta elegans* |  |  | x |  |
|  |  |  |  |  | *Parasagitta setosa* | x cf. |  |  |  |
|  |  |  |  | *Pseudosagitta* | *Pseudosagitta lyra* | x |  |  |  |
|  |  |  |  | *Sagitta* |  |  |  | x | x |
|  |  |  | Sagittidae |  |  |  |  | x |  |
|  |  |  |  |  |  |  |  | x |  |
| Arthropoda | Branchiopoda | Ctenopoda | Sididae | *Penilia* | *Penilia avirostris* | x |  | x | x |
|  |  | Diplostraca |  |  |  |  |  | x |  |
|  |  | Onychopoda | Podonidae | *Evadne* | *Evadne nordmanni* |  |  | x | x |
|  |  |  |  |  | *Evadne spinifera* |  |  | x | x |
|  |  |  |  | *Pleopis* | *Pleopis polyphemoides* | x |  |  | x |
|  |  |  |  | *Podon* | *Podon intermedius* |  |  |  | x |
|  |  |  |  | *Podon* |  |  |  | x |  |
|  |  |  |  | *Pseudevadne* | *Pseudevadne tergestina* |  |  |  | x |
|  |  |  | Podonidae |  |  |  |  | x | x |
|  | Copepoda | Calanoida | Acartiidae | *Acartia* | *Acartia clausi* | x | x | x | x |
|  |  |  |  | *Acartia* |  |  |  | x | x |
|  |  |  | Calanidae | *Calanus* | *Calanus helgolandicus* |  |  | x | x |
|  |  |  |  | *Calanus* |  | x |  | x |  |
|  |  |  |  | *Nannocalanus* | *Nannocalanus minor* | x |  |  |  |
|  |  |  |  | *Neocalanus* |  |  |  | x |  |
|  |  |  | Centropagidae | *Centropages* | *Centropages hamatus* |  |  | x |  |
|  |  |  |  |  | *Centropages ponticus* | x cf. |  |  | x |
|  |  |  |  |  | *Centropages typicus* | x | x cf. | x | x |
|  |  |  |  | *Isias* | *Isias clavipes* |  |  | x | x |
|  |  |  | Clausocalanidae | *Clausocalanus* | *Clausocalanus arcuicornis* |  |  |  | x |
|  |  |  |  |  | *Clausocalanus furcatus* | x |  | x | x |
|  |  |  |  |  | *Clausocalanus jobei* | x |  |  | x |
|  |  |  |  |  | *Clausocalanus parapergens* | x |  | x cf. |  |
|  |  |  |  |  | *Clausocalanus paululus* | x |  |  |  |
|  |  |  |  |  | *Clausocalanus pergens* | x |  | x | x |
|  |  |  |  | *Clausocalanus* |  | x |  | x | x |
|  |  |  |  | *Ctenocalanus* | *Ctenocalanus vanus* | x | x |  | x |
|  |  |  |  | *Ctenocalanus* |  |  |  | x | x |
|  |  |  | Euchaetidae | *Euchaeta* |  | x |  | x cf. |  |
|  |  |  | Lucicutiidae | *Lucicutia* |  | x |  |  |  |
|  |  |  | Metridinidae | *Pleuromamma* | *Pleuromamma gracilis* | x |  |  |  |
|  |  |  | Paracalanidae | *Calocalanus* |  |  |  | x | x |
|  |  |  |  | *Calocalanus* | *Calocalanus pavo* | x |  |  |  |
|  |  |  |  |  | *Calocalanus plumulosus* |  |  |  | x |
|  |  |  |  |  | *Calocalanus styliremis* | x cf. |  |  | x |
|  |  |  |  | *Paracalanus* | *Paracalanus indicus* | x |  |  |  |
|  |  |  |  |  | *Paracalanus nanus* | x |  |  | x |
|  |  |  |  |  | *Paracalanus parvus* | x | x | x | x |
|  |  |  |  |  | *Paracalanus quasimodo* | x |  |  |  |
|  |  |  |  | *Paracalanus* |  | x |  | x | x |
|  |  |  | Pseudodiaptomidae | *Pseudodiaptomus* | *Pseudodiaptomus marinus* |  |  | x | x |
|  |  |  | Scolecitrichidae | *Scolecithricella* | *Scolecithricella sp.* | x |  |  |  |
|  |  |  |  |  | *Scolecithrix pygmaea* |  |  |  | x |
|  |  |  | Spinocalanidae | *Mimocalanus* | *Mimocalanus aff. cultrifer* | x |  |  |  |
|  |  |  | Temoridae | *Temora* | *Temora discaudata* |  |  | x |  |
|  |  |  |  |  | *Temora longicornis* |  |  |  | x |
|  |  |  |  |  | *Temora sp.* |  |  | x cf. |  |
|  |  |  |  |  | *Temora stylifera* | x |  | x | x |
|  |  |  |  |  | *Temora turbinata* |  |  | x |  |
|  |  |  |  | *Temoropia* | *Temoropia mayumbaensis* | x cf. |  |  |  |
|  |  |  |  |  |  | x |  | x | x |
|  |  | Canuelloida | Longipediidae | *Longipedia* |  |  |  | x |  |
|  |  | Cyclopoida | Corycaeidae | *Ditrichocorycaeus* | *Ditrichocorycaeus anglicus* | x cf. | x |  |  |
|  |  |  |  |  | *Ditrichocorycaeus brehmi* |  |  |  | x* |
|  |  |  |  | *Ditrichocorycaeus* |  |  |  | x |  |
|  |  |  |  | *Farranula* |  | x |  | x |  |
|  |  |  |  | *Onychocorycaeus* | *Onychocorycaeus giesbrechti* |  |  |  | x* |
|  |  |  | Corycaeidae |  |  |  |  | x | x |
|  |  |  | Oithonidae | *Oithona* | *Oithona longispina* |  |  |  | x |
|  |  |  |  |  | *Oithona nana* |  |  | x | x |
|  |  |  |  |  | *Oithona plumifera* |  |  | x | x |
|  |  |  |  |  | *Oithona similis* | x | x cf. | x | x |
|  |  |  |  | *Oithona* |  |  |  | x | x |
|  |  |  | Oithonidae |  |  |  |  | x | x |
|  |  |  | Oncaeidae | *Monothula* | *Monothula subtilis* |  |  |  | x* |
|  |  |  |  | *Oncaea* | *Oncaea curta* | x |  | x | x |
|  |  |  |  |  | *Oncaea media* |  |  |  | x |
|  |  |  |  |  | *Oncaea mediterranea* |  |  |  | x |
|  |  |  |  |  | *Oncaea venusta venusta* |  |  |  | x |
|  |  |  |  |  | *Oncaea waldemari* | x cf. |  |  |  |
|  |  |  |  | *Oncaea* |  |  |  | x | x |
|  |  |  |  |  |  |  |  | x |  |
|  |  | Harpacticoida | Clytemnestridae |  |  |  |  | x |  |
|  |  |  | Peltidiidae | *Goniopsyllus* |  |  |  |  | x |
|  |  |  | Ectinosomatidae | *Microsetella* | *Microsetella norvegica* |  |  | x |  |
|  |  |  |  | *Microsetella* |  |  |  | x | x |
|  |  |  | Tachidiidae | *Euterpina* | *Euterpina acutifrons* |  |  | x | x |
|  |  |  |  |  |  |  |  | x | x |
|  |  | Monstrilloida | Monstrillidae | *Cymbasoma* |  |  |  | x | x |
|  |  |  |  |  |  | x | x | x | x |
|  | Malacostraca | Amphipoda | Corophiidae | *Grandidierella* | *Grandidierella japonica* | x |  |  |  |
|  |  |  | Ischyroceridae | *Jassa* | *Jassa slatteryi* | x |  | x |  |
|  |  |  |  |  |  |  |  | x | x |
|  |  | Decapoda | Carcinidae | *Carcinus* | *Carcinus maenas* |  | x |  |  |
|  |  |  | Paguridae |  |  |  |  | x cf. |  |
|  |  |  | Pandalidae | *Plesionika* | *Plesionika martia* | x |  |  |  |
|  |  |  | Parthenopidae | *Leiolambrus* |  |  |  | x |  |
|  |  |  | Polybiidae | *Liocarcinus* | *Liocarcinus maculatus* |  |  | x |  |
|  |  |  | Porcellanidae | *Pisidia* |  |  |  | x | x |
|  |  |  |  |  |  |  |  | x | x |
|  |  | Euphausiacea | Euphausiidae | *Euphausia* |  | x |  |  |  |
|  |  |  | Euphausiidae | *Thysanopoda* |  | x |  |  |  |
|  |  | Mysida | Mysidae | *Acanthomysis* |  |  |  | x |  |
|  |  |  |  |  |  |  |  | x |  |
|  | Ostracoda | Halocyprida | Halocyprididae | *Conchoecia* | *Conchoecia sp.* |  |  | x |  |
|  |  |  |  | *Mikroconchoecia* | *Mikroconchoecia sp.* | x |  |  |  |
|  |  |  |  |  |  |  |  | x | x |
|  | Thecostraca |  |  |  |  |  |  | x | x |
| Echinodermata | Asteroidea | Forcipulatida | Asteriidae | *Diplasterias* |  |  |  | x |  |
|  |  |  | Pedicellasteridae |  |  | x |  |  |  |
|  |  |  |  |  |  |  |  | x | x |
|  | Echinoidea | Camarodonta | Echinidae | *Paracentrotus* | *Paracentrotus lividus* |  | x |  |  |
|  |  |  |  |  |  |  |  |  | x |
|  | Holothuroidea | Holothuriida | Holothuriidae | *Holothuria* | *Holothuria atra* | x |  |  |  |
|  |  |  | Holothuriidae |  |  |  |  | x |  |
|  |  |  |  |  |  |  |  | x | x |
|  | Ophiuroidea | Amphilepidida | Amphiuridae | *Amphiura* |  |  |  | x |  |
|  |  |  |  |  |  |  |  | x | x |
|  |  |  |  |  |  |  |  | x | x |
| Chordata | Appendicularia | Copelata | Fritillariidae | *Fritillaria* | *Fritillaria borealis* |  |  | x |  |
|  |  |  | Fritillariidae | *Fritillaria* |  |  |  | x | x |
|  |  |  |  | *Oikopleura* | *Oikopleura (Vexillaria) dioica* |  | x | x |  |
|  |  |  |  |  | *Oikopleura fusiformis* |  |  | x |  |
|  |  |  |  |  | *Oikopleura longicauda* |  |  | x |  |
|  |  |  |  | *Oikopleura* |  |  |  | x | x |
|  |  |  | Oikopleuridae |  |  |  |  | x |  |
|  |  |  |  |  |  | x |  |  |  |
|  | Ascidiacea |  |  |  |  |  |  |  | x |
|  | Teleostei | Callionymiformes | Callionymidae | *Callionymus* | *Callionymus lyra* |  |  | x |  |
|  |  | Clupeiformes | Clupeidae | *Alosa* | *Alosa alosa* |  |  | x |  |
|  |  |  |  | *Sprattus* | *Sprattus sprattus* |  |  | x |  |
|  |  | Cypriniformes | Cyprinidae | *Rutilus* | *Rutilus rutilus* |  | x |  |  |
|  |  | Gobiiformes | Gobiidae | *Awaous* | *Awaous commersoni* | x |  |  |  |
|  |  | Pleuronectiformes | Soleidae | *Solea* | *Solea Solea* | x |  | x |  |
|  |  |  |  |  |  |  |  | x | x |
|  | Thaliacea | Copelata | Doliolidae |  |  |  |  |  | x |
